# Supplementary material for: Joint modeling of cellular heterogeneity and condition effects with scPCA in single-cell RNA-seq
Source: Commun Biol. 2026 Feb 4;9:492. doi: 10.1038/s42003-026-09651-6 (PMC13057030; doi:10.1038/s42003-026-09651-6)
Supplement: Supplementary file 2 — Supplementary Information [file 42003_2026_9651_MOESM2_ESM.pdf]

Joint Modeling of Cellular Heterogeneity and Condition Effects with scPCA in Single-Cell RNA-Seq

Harald Vöhringer[1-4]

[1] European Molecular Biology Laboratory (EMBL), Meyerhofstrasse 1, 69117 Heidelberg, Germany
[2] Molecular Medicine Partnership Unit (MMPU), Heidelberg, Germany
[3] Department of Medicine V, Hematology, Oncology and Rheumatology, Im Neuenheimer Feld 410, Heidelberg, Germany
[4] Department of Hematology and Oncology, University Hospital Düsseldorf, Moorenstrasse 5, 40225 Düsseldorf, Germany

Supplementary Methods

The scPCA model

The scPCA factorisation requires a multi-condition single-cell expression count matrix $\mathbf{X} \in \mathbb{N}_0^{C \times G}$ for C cells and G genes, a design matrix $\mathbf{D} \in \{0,1\}^{C \times Q}$ that encodes a conditioning variable with Q levels, and an optional indicator matrix $\mathbf{B} \in \{0,1\}^{C \times P}$ that may adjust for condition-specific mean gene expression for P groups of cells (usually $P=1$ or $P=Q$, i.e. scPCA fits a global or condition-specific mean offset(s) respectively) as inputs. We model the elements of the expression count matrix $\mathbf{X} = \{x_{cg}\}$ for cell $c = 1, \ldots, C$ and gene $g = 1, \ldots, G$ as negative binomial distributed, given an unobserved gene expression level μ_{cg} and a gene-specific over-dispersion α_g

$$x_{cg} \sim \mathrm{NB}(\mu_{cg}, \alpha_g). \tag{1}$$

In scPCA, we decompose the unobserved gene expression as a linear function of the conditioning variable encoded in $\mathbf{D}$. Denote the entries of the design and indicator matrix $\mathbf{D} = \{d_{cq}\}$ and $\mathbf{B} = \{b_{cp}\}$ for level $q = 1, \ldots, Q$ and group $p = 1, \ldots, P$ respectively, and let $l_c = \log \sum_{g=1}^{G} x_{cg}$ denote a cell-specific fixed size factor (total number of gene counts). Then,

$$\log \mu_{cg} = \underbrace{l_c}_{\text{Size factor}} + \underbrace{\left(\sum_{p=1}^{P} b_{cp} v_{pg} \right)_{cg}}_{\text{Intercept term}} + \underbrace{\left(\sum_{q=1}^{Q} d_{cq} \cdot \sum_{f=1}^{F} z_{cf} w_{fgq} \right)_{cg}}_{\text{Factor model}} \tag{2}$$

where w_{fgq} represents the entries of a loading weight tensor $\mathbf{W} \in \mathbb{R}^{F \times G \times Q}$ consisting of $f = 1, \ldots, F$ components, z_{cf} the elements a factor weight matrix $\mathbf{Z} \in \mathbb{R}^{C \times F}$ containing the coordinates of each cell with respect to $\mathbf{W}$, and v_{pg} the entries of a mean offset $\mathbf{V} \in \mathbb{R}^{P \times G}$ matrix.

Model inputs and parameters

The expression count matrix

In the following, we will restrict our discussion to a single conditioning variable with levels $q = 1, \ldots, Q$. To jointly model multi-condition single-cell data using scPCA, we require the user to decide on a reference level for comparison. For example, in the common control-treatment setting, the cells of the control condition represent a natural choice. In general, the expression count matrix may take the form

$$\mathbf{X} = \begin{bmatrix} \mathbf{X}^1 \\ \mathbf{X}^2 \\ \mathbf{X}^3 \\ \vdots \\ \mathbf{X}^Q \end{bmatrix} \tag{3}$$

where $\mathbf{X} \in \mathbb{N}_0^{C \times G}$ for $C = \sum_{q=1}^{Q} C_q$ cells and G genes, and $\mathbf{X}^q \in \mathbb{N}_0^{C_q \times G}$ denotes the single-cell expression count matrices from the control ($q = 1$) and each treatment level ($q = 2, \ldots, Q$).

The loading weight tensor **W**

In order to account for the conditioning variable, we introduce for each level a $F \times G$ gene loading weights, giving rise to a $F \times G \times Q$ sized loading tensor $\mathbf{W}$. Another way to conceptualize this is to view $\mathbf{W}$ as a stacked array of Q loading matrices

$$\mathbf{W} = \left[\mathbf{W}^1; \mathbf{W}^2; \ldots; \mathbf{W}^Q\right] \tag{4}$$

where each $\mathbf{W}^q = \{w_{fgq}\}$ for $q = 1, \ldots, Q$ represents a $F \times G$ matrix.

The loading weight design matrix **D**

To inform the factorisation framework about the conditioning variable, we utilize full-column rank design matrices. For the expression count matrix in Eq (3), $\mathbf{D}$ takes the form

$$\mathbf{D} = \begin{bmatrix} \mathbf{1}_{C_1} & \mathbf{0}_{C_1} & \mathbf{0}_{C_1} & \ldots & \mathbf{0}_{C_1} \\ \mathbf{1}_{C_2} & \mathbf{1}_{C_2} & \mathbf{0}_{C_2} & \ldots & \mathbf{0}_{C_2} \\ \mathbf{1}_{C_3} & \mathbf{0}_{C_3} & \mathbf{1}_{C_3} & \ldots & \mathbf{0}_{C_3} \\ \vdots & \vdots & \vdots & \ddots & \vdots \\ \mathbf{1}_{C_Q} & \mathbf{0}_{C_Q} & \mathbf{0}_{C_Q} & \ldots & \mathbf{1}_{C_Q} \end{bmatrix} \in \mathbb{R}^{C \times Q} \tag{5}$$

where $\mathbf{1}_{C_q}$ and $\mathbf{0}_{C_q}$ represent column vectors of length C_q for $q = 1, \ldots, Q$ with ones and zeros, respectively. Note that the first column of $\mathbf{D}$ encodes the reference level and contains only ones. All other columns $2, \ldots, Q$ contain ones in rows where $\mathbf{X}$ (Eq. (3)) harbors cells of the respective condition and otherwise zeros. Statistically inclined readers will recognise that the form of $\mathbf{D}$ is reminiscent of design matrices employed in multiple linear regression (MLR) models with indicator variables. Similar to MLR models, where the design matrices parameterise regression coefficients, scPCA leverages $\mathbf{D}$ to sum slices of the loading tensor $\mathbf{W}^q$ (see Eq. (4)) in a condition-dependent manner.

The mean offset matrix **V**

In scPCA, we directly model single-cell expression data in count space (Eq. (1)), thereby evading the need to transform the data prior to the analysis. However, in order to find the condition-specific bases it is mandatory to center the data[1]. For this reason, we introduce the parameter $\mathbf{V}$ which captures the mean expression of each gene.

The mean offset indicator matrix **B**

We enable parameterizing $\mathbf{V}$ by providing an optional indicator matrix $\mathbf{B}$. We found that a global mean offset suffices for most multi-condition datasets. In this case, $\mathbf{B}$ reduces to a column vector of ones. However, if the experimental intervention causes shifts in mean gene expression, distinct mean offsets for each condition are crucial to ensure alignment of the bases. Then, $\mathbf{B}$ takes the form of an indicator matrix

$$\mathbf{B} = \begin{bmatrix} \mathbf{1}_{C_1} & \mathbf{0}_{C_1} & \mathbf{0}_{C_1} & \cdots & \mathbf{0}_{C_1} \\ \mathbf{0}_{C_2} & \mathbf{1}_{C_2} & \mathbf{0}_{C_2} & \cdots & \mathbf{0}_{C_2} \\ \mathbf{0}_{C_3} & \mathbf{0}_{C_3} & \mathbf{1}_{C_3} & \cdots & \mathbf{0}_{C_3} \\ \vdots & \vdots & \vdots & \ddots & \vdots \\ \mathbf{0}_{C_Q} & \mathbf{0}_{C_Q} & \mathbf{0}_{C_Q} & \cdots & \mathbf{1}_{C_Q} \end{bmatrix}. \tag{6}$$

Finally, we compute a cell-specific size factor $l_c = \log \sum_{g=1}^{G} x_{cg}$ which accounts for technical differences in sequencing depth between cells.

Model Parameterisation and Intuition

Consider a conditioning variable with $Q = 2$ levels. For example, we might be interested in jointly modeling single-cell data from a control (ctl) and treatment (trt) experiment comprising C_1 ctl and C_2 trt cells, respectively. The concatenated transcript count matrix $\mathbf{X} \in \mathbb{N}_0^{C \times G}$ where $C = C_1 + C_2$ may then take the form

$$\mathbf{X} = \begin{bmatrix} \mathbf{X}^{\text{ctl}} \\ \mathbf{X}^{\text{trt}} \end{bmatrix}$$

where $\mathbf{X}^{\text{ctl}} \in \mathbb{N}_0^{C_1 \times G}$ and $\mathbf{X}^{\text{trt}} \in \mathbb{N}_0^{C_2 \times G}$ contain the expression data from ctl and trt cells, respectively.

Choosing the ctl condition as reference, we consider the following design and indicator matrices

$$\mathbf{D} = \begin{bmatrix} \mathbf{1}_{C_1} & \mathbf{0}_{C_1} \\ \mathbf{1}_{C_2} & \mathbf{1}_{C_2} \end{bmatrix} \quad \text{and} \quad \mathbf{B} = \begin{bmatrix} \mathbf{1}_{C_1} & \mathbf{0}_{C_1} \\ \mathbf{0}_{C_2} & \mathbf{1}_{C_2} \end{bmatrix}$$

where $\mathbf{1}_{C_q}$ and $\mathbf{0}_q$ represent column vectors of length C_q for $q = \{1, 2\}$ with ones and zeros, respectively. Denote $\mathbf{d}_c$ as the cth row vector of $\mathbf{D}$ (and similarly for all other matrices). Note that a cell i from the ctl condition is encoded with the row vectors $\mathbf{d}_i = [1, 0]$ and $\mathbf{b}_i = [1, 0]$, respectively. Conversely, a cell j from the trt condition is represented by row vectors in the form of $\mathbf{d}_j = [1, 1]$ and $\mathbf{b}_j = [0, 1]$, respectively.

To understand how the design and indicator matrices $\mathbf{D}$ and $\mathbf{B}$ parameterise the model, we will now focus on the factor model term $\mathbf{S}$ and intercept term $\mathbf{T}$ separately (compare Eq. (2)), i.e.

$$\log \mu_{cg} = l_c + \underbrace{\left(\sum_{p=1}^{P} b_{cp} v_{pg} \right)_{cg}}_{\mathbf{T}} + \underbrace{\left(\sum_{q=1}^{Q} d_{cq} \cdot \sum_{f=1}^{F} z_{cf} w_{fgq} \right)_{cg}}_{\mathbf{S}} . \tag{7}$$

The parameterisation of the loading tensor **W**

We start by expanding the term $\mathbf{S}$ of Eq. (7) for a particular row c

$$\mathbf{s}_c = d_{c1} \cdot \textstyle\sum_{f=1}^{F} z_{cf} w_{fg1} + d_{c2} \cdot \textstyle\sum_{f=1}^{F} z_{cf} w_{fg2}$$

and explicitly write out the case for a cell $c = i$ belonging to the control group (i.e. $\mathbf{d}_i = [1, 0]$)

$$\begin{aligned} \mathbf{s}_i &= 1 \cdot \sum_{f=1}^{F} z_{if} w_{fg1} + 0 \cdot \sum_{f=1}^{F} z_{if} w_{fg2} \\ &= \sum_{f=1}^{F} z_{if} w_{fg1} \\ &= \mathbf{z}_i \mathbf{W}^1 = \mathbf{z}_i \mathbf{W}^{\text{ctl}} \end{aligned}$$

and likewise for a treated cell $c = j$ (i.e. $\mathbf{d}_j = [1, 1]$)

$$\begin{aligned} \mathbf{s}_j &= 1 \cdot \sum_{f=1}^{F} z_{jf} w_{fg1} + 1 \cdot \sum_{f=1}^{F} z_{jf} w_{fg2} \\ &= \sum_{f=1}^{F} z_{jf} \left(w_{fg1} + w_{fg2} \right) \\ &= \mathbf{z}_j \left(\mathbf{W}^1 + \mathbf{W}^2 \right) = \mathbf{z}_j \left(\mathbf{W}^{\text{ctl}} + \mathbf{W}^2 \right) = \mathbf{z}_j \mathbf{W}^{\text{trt}}. \end{aligned}$$

We find that the design matrix instructs the model to sum slices of the loading tensor in a cell/condition-dependent manner. Note that $\mathbf{D}$'s first column contains ones to designate the user-specified reference group. As a consequence, the bases for each other experimental condition encoded in $\mathbf{D}$ is a linear combination of the first slice $\mathbf{W}^1$ which represents the loading matrix of the ctl group ($\mathbf{W}^{\text{ctl}}$). We stress the importance of expressing the loading matrices of an experimental condition with respect to the specified reference because it allows the model to fit aligned components.

The parameterisation of the mean offset matrix $\mathbf{V}$

We now focus on the intercept term of Eq. (7). We observed that supplying an indicator matrix is optional for most single-cell datasets, in which case the $\mathbf{B}$ reduces to a vector of ones $\mathbf{1}_C$ and global mean offsets are inferred. However, in cases when it is reasonable to assume that there are substantial deviations in mean expression across conditions, it may be mandatory to center gene expression in a condition-specific manner. Then $\mathbf{B}$ takes the form of an indicator matrix (see Eq. (6)).

We first expand term $\mathbf{T}$ from Eq. (7) for a particular row c

$$\mathbf{t}_c = b_{c1} \cdot v_{1g} + b_{c2} \cdot v_{2g}$$

and explicitly write out the case for a cell $c = i$ belonging to the ctl group (i.e. $\mathbf{b}_i = [1, 0]$), then

$$\begin{aligned} \mathbf{t}_i &= 1 \cdot v_{1g} + 0 \cdot v_{2g} \\ &= \mathbf{v}_1 = \mathbf{v}^{\text{ctl}} \end{aligned}$$

and likewise for a cell $c = j$ that is member of the trt group (i.e. $\mathbf{b}_j = [0, 1]$) reduces to

$$\begin{aligned} \mathbf{t}_j &= 0 \cdot v_{1g} + 1 \cdot v_{2g} \\ &= \mathbf{v}_2 = \mathbf{v}^{\text{trt}}. \end{aligned}$$

Taken together, we find that the intercept design matrix $\mathbf{B}$ effectively extracts distinct rows from $\mathbf{V}$ in a cell-specific manner, thereby allowing to model the mean across the P groups defined in the indicator matrix $\mathbf{B}$.

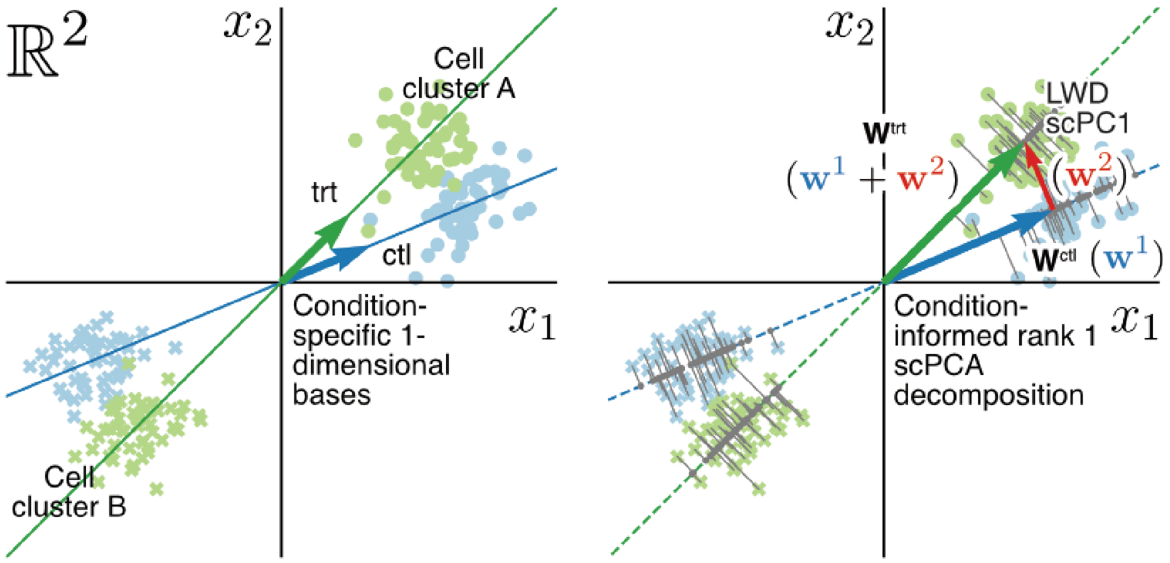

Loading weight differences (LWDs)

The Loading Weight Difference (LWD) is defined as the (elementwise) matrix difference between conditions (here the treatment and control directions). In this example, it corresponds exactly to

$$\mathrm{LWD}^{\mathrm{trt}} = \mathbf{W}^{\mathrm{trt}} - \mathbf{W}^{\mathrm{ctl}} = \mathbf{W}^2$$

Conceptually, LWDs quantify the magnitude and direction of the perturbation-induced shift in the latent space, capturing how each factor is altered under a given condition. In practice, the resulting LWD matrix highlights which genes are most affected by a perturbation along specific latent components. These values can be ranked to identify perturbation-responsive genes, and are used for downstream analyses such as gene set enrichment.

Disentangling Batch and Condition effects

The flexible factorization framework of scPCA enables it to disentangle batch effects from biological conditions in scRNA-seq data. However, to successfully separate batch and perturbation effects, scPCA requires a balanced experimental design—that is, all combinations of batch and perturbation must be represented. This design is crucial to ensure the model can statistically identify and distinguish technical variation from true biological signals.

To exemplify how scPCA addresses this challenge, we discuss two illustrative examples using a simulated 2-dimensional toy dataset. These examples demonstrate intuitively how scPCA disentangles batch and perturbation effects under two different assumptions:

1. In the first scenario, batch effects manifest as global shifts in mean expression, while perturbations correspond to rotations in latent space.

2. In the second scenario, both batch and perturbation effects arise from rotations.

While these examples focus on shifts in location and rotations, we note that the scPCA framework is, in principle, capable of capturing a broad range of affine transformations—including shearing and scaling—through its condition- and batch-specific parameterization of the loading tensor $\mathbf{W}$ and the mean offset matrix $\mathbf{V}$.

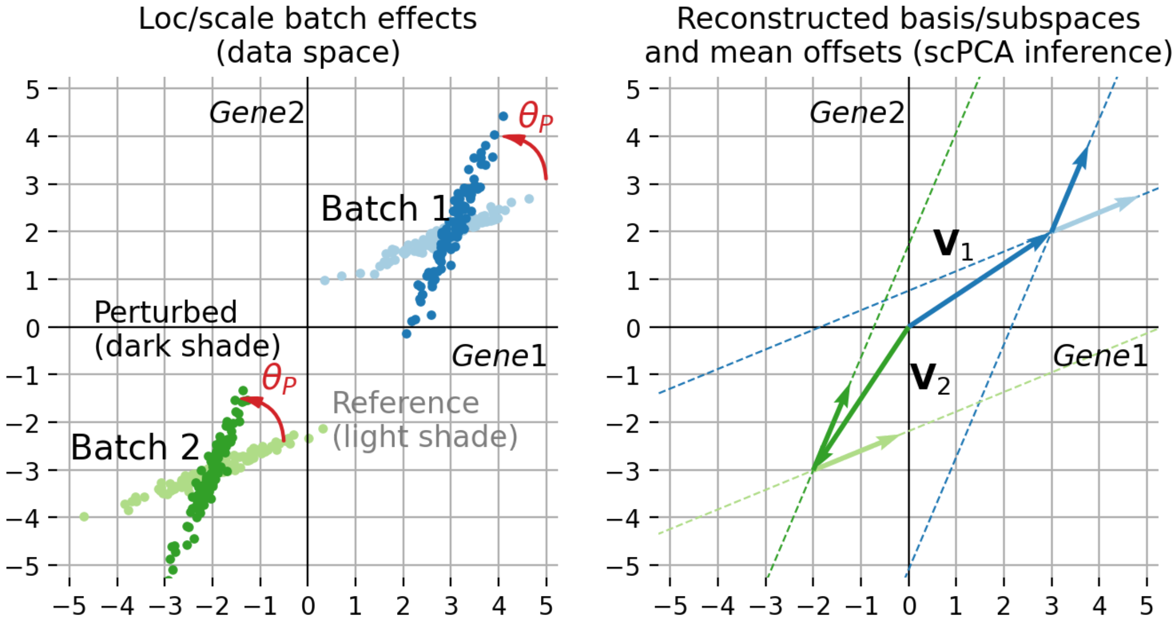

In the first example, perturbations are represented as rotations (θ_P) in data space, while batch effects result from shifts in location. In real single-cell datasets, such batch effects often present as global shifts in average gene expression, for example due to differences in sequencing protocols or platforms. scPCA addresses this by allowing the user to specify a mean offset matrix $\mathbf{V}$, which accounts for batch-specific shifts. This enables the model to recover an appropriate basis for reference condition (right panel) while simultaneously learning appropriate transformations to align the perturbed data (left panel). As a result, scPCA captures consistent biological variation while preserving underlying cell-type structure across batches.

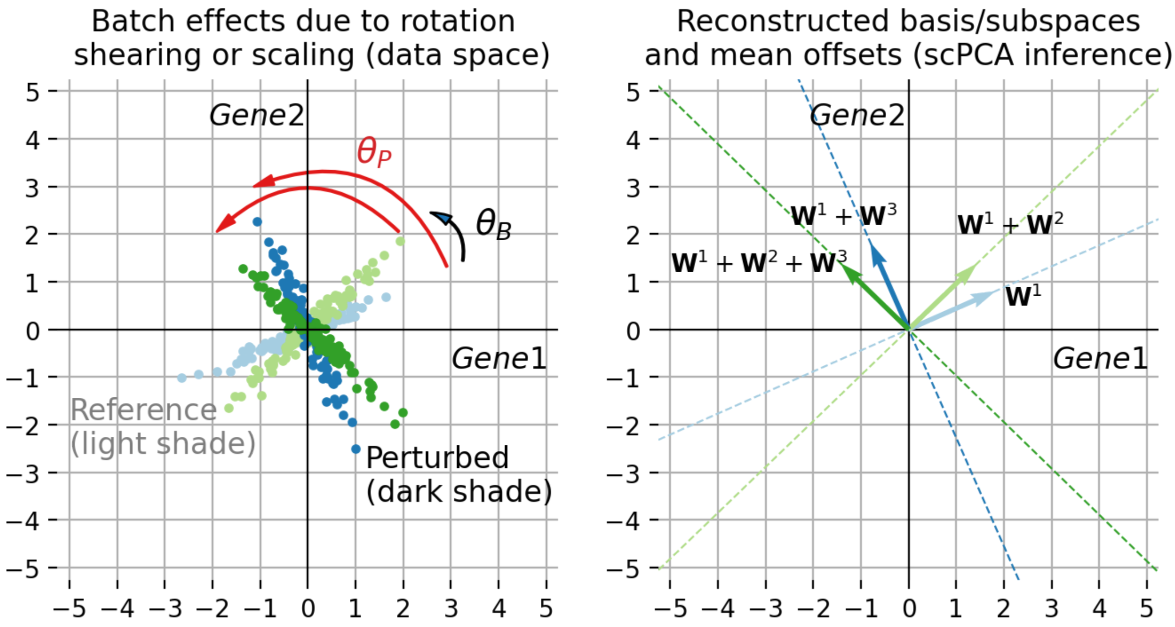

The second example represents a more complex scenario in which both batch (θ_B) and perturbation effects (θ_P) arise from rotations. To disentangle these sources of variation, scPCA relies on an appropriate loading design formula, such as batch + design, which instructs the model to represent each batch–condition combination with a distinct linear combination of the loading tensor. This formulation allows scPCA to accurately capture the shared perturbation direction (encoded in the loading tensor slice W^3) while learning batch-specific linear transformations (loading tensor slice W^2). As a result, the model successfully separates biological perturbation effects from batch-induced distortions in the data space.

Implementation and Inference

We express the scPCA in terms of a hierarchical Bayesian model. The generative model may be expressed as

$$
\begin{aligned}
x_{cg} \mid \mu_{cg}, \alpha_g &\sim \mathrm{NB}\left(\mu_{cg}, \alpha_g\right) \\
\log \mu_{cg} &= l_c + \left(\sum_{p=1}^{P} b_{cp} v_{pg}\right)_{cg} + \left(\sum_{q=1}^{Q} d_{cq} \cdot \sum_{f=1}^{F} z_{cf} w_{fgq}\right)_{cg} \\
z_{cf} &\sim \mathcal{N}(0, 0.1) \\
w_{fgq} &\sim \mathcal{N}(0, 1) \\
v_{pg} &\sim \mathcal{N}(0, 1) \\
\alpha_g^{-1} &\sim \mathrm{Exp}(\beta) \\
\beta &\sim \mathrm{Gamma}(3, 0.01).
\end{aligned}
\tag{4}
$$

Importantly, scPCA models the observed count data using a Negative Binomial (NB) likelihood. We opted for the NB distribution over a zero-inflated NB model because recent studies indicate that zero-inflation is unnecessary for droplet-based scRNA-seq data[2]. The

NB distribution effectively captures essential count properties such as overdispersion and the mean–variance relationship, while avoiding the added complexity of zero-inflated models. This choice enables more efficient and stable gradient-based optimization, which is crucial for scalable single-cell data analysis.

We choose Normal distributions as priors for the latent variables $\mathbf{W}$, $\mathbf{V}$ and $\mathbf{Z}$ of the model. Observe that we posit a scale of 0.1 on the prior for $\mathbf{Z}$ thereby encouraging the model to capture spectral differences in gene expression across conditions in the loading tensor $\mathbf{W}$ rather than to explain them with additional factors.

We use a containment prior to model the overdispersion α_g of each gene. Apriori the model assumes equidispersed (poissonian) counts by placing most probability mass on large α_g. Note that we tightly fix the mean exponential rate β around 3 with a standard deviation of 0.01.

Using matrix notation we may express the priors of the model as follows

$$\begin{aligned}
p(\beta) &= \text{Gamma}(\beta; 3, 0.01) \\
p\left(\boldsymbol{\alpha}^{-1} \mid \beta\right) &= \prod_{g=1}^{G} \text{Exp}\left(\alpha_g^{-1} \mid \beta\right) \\
p(\mathbf{V}) &= \prod_{p=1}^{P} \prod_{g=1}^{G} \mathcal{N}\left(v_{pg}; 0, 1\right) = \prod_{p=1}^{P} \mathcal{N}\left(\mathbf{v}_p; \mathbf{0}_G, \mathbf{I}_G\right) \\
p(\mathbf{W}) &= \prod_{q=1}^{Q} \prod_{f=1}^{F} \prod_{g=1}^{G} \mathcal{N}\left(w_{fgq}; 0, 1\right) = \prod_{q=1}^{Q} \prod_{f=1}^{F} \mathcal{N}\left(\mathbf{w}_f^q; \mathbf{0}_G, \mathbf{I}_G\right) \\
p(\mathbf{Z}) &= \prod_{c=1}^{C} \prod_{f=1}^{F} \mathcal{N}\left(z_{cf}; 0, 0.1\right) = \prod_{c=1}^{C} \mathcal{N}\left(\mathbf{z}_c; \mathbf{0}_F, 0.1 \cdot \mathbf{I}_F\right)
\end{aligned}$$

where $\mathbf{0}_A$ represents a A-dimensional vector of zeros and $\mathbf{I}_A$ an $A \times A$ identity matrix. Together with the likelihood of the data

$$p\left(\mathbf{X} \mid \mathbf{W}, \mathbf{Z}, \mathbf{V}, \boldsymbol{\alpha}^{-1}\right) = \prod_{c=1}^{C} \prod_{g=1}^{G} \text{NB}\left(x_{cg} \mid \mu_{cg}, \alpha_g\right)$$

we derive the joint distribution

$$\begin{aligned}
p\left(\mathbf{X}, \mathbf{W}, \mathbf{Z}, \mathbf{V}, \boldsymbol{\alpha}^{-1}, \beta\right) =& p(\beta) p\left(\boldsymbol{\alpha}^{-1} \mid \beta\right) p(\mathbf{W}) p(\mathbf{V}) p(\mathbf{Z}) p\left(\mathbf{X} \mid \mathbf{W}, \mathbf{Z}, \mathbf{V}, \boldsymbol{\alpha}^{-1}\right) \\
=& \text{Gamma}(\beta; 3, 0.01) \\
& \times \prod_{p=1}^{P} \mathcal{N}\left(\mathbf{v}_p; \mathbf{0}_G, \mathbf{I}_G\right) \\
& \times \prod_{q=1}^{Q} \prod_{f=1}^{F} \mathcal{N}\left(\mathbf{w}_f^q; \mathbf{0}_G, \mathbf{I}_G\right) \\
& \times \prod_{g=1}^{G} \text{Exp}\left(\alpha_g^{-1}; \beta\right) \prod_{c=1}^{C} \text{NB}\left(x_{cg} \mid \mu_{cg}, \alpha_g\right) \mathcal{N}\left(\mathbf{z}_c; \mathbf{0}_F, 0.1 \cdot \mathbf{I}_F\right).
\end{aligned}$$

We use stochastic variational inference in order the determine the posterior distribution of the latent variables of the model $p(\boldsymbol{\theta}, \boldsymbol{\alpha}^{-1}, \beta|\mathbf{X})$ where $\boldsymbol{\theta} = \{\mathbf{W}, \mathbf{V}, \mathbf{Z}\}$. Variational methods require us to define a surrogate posterior $q\left(\boldsymbol{\theta}, \boldsymbol{\alpha}^{-1}, \beta; \boldsymbol{\phi}\right)$ which serves as an approximation to the true posterior. For this reason, we posit a mean-field approximation comprising normal and log normal distributions

$$q\left(\boldsymbol{\theta}, \boldsymbol{\alpha}^{-1}, \beta; \boldsymbol{\phi}\right) = \log \mathrm{N}\left(\beta; m_1, s_1\right) \prod_{k=2}^{G+1} \log \mathrm{N}\left(\alpha_g^{-1}; m_k, s_k\right) \prod_{k=G+2}^{K} \mathcal{N}\left(\theta_k; m_k, s_k\right)$$

where $\phi = (m_1, \ldots, m_K, s_1, \ldots, s_K)$ concatenates the mean and standard deviation of each Gaussian factor, and $K = 1 + G + P \times G + F \times G \times Q + C \times F$ represents the total number of parameters in the model.

We aim to find the variational parameters ϕ that minimize the KL divergence between surrogate and true posterior $\mathrm{KL}\left(q\left(\boldsymbol{\theta}, \boldsymbol{\alpha}^{-1}, \beta; \boldsymbol{\phi}\right) \| p\left(\boldsymbol{\theta}, \boldsymbol{\alpha}^{-1}, \beta \mid \mathbf{X}\right)\right)$. However, since $p\left(\boldsymbol{\theta}, \boldsymbol{\alpha}^{-1}, \beta \mid \mathbf{X}\right)$ is unknown, we instead use the evidence lower bound (ELBO)[3]

$$\mathcal{L}(\boldsymbol{\phi}) = \mathbb{E}_{q(\boldsymbol{\theta}, \boldsymbol{\alpha}^{-1}, \beta)}\left[\log p\left(\mathbf{X}, \boldsymbol{\theta}, \boldsymbol{\alpha}^{-1}, \beta\right) - \log q\left(\boldsymbol{\theta}, \boldsymbol{\alpha}^{-1}, \beta; \boldsymbol{\phi}\right)\right]$$

which is equivalent to the KL divergence up to a constant. We use a gradient descent-based method (black box variational inference) to solve the optimisation problem

$$\phi^* = \arg\max_{\phi} \mathcal{L}(\phi)$$

which requires us to estimate the gradients of the expectation in Eq. (5) with respect to the variational parameters ϕ. In practice this may be achieved using Monte Carlo integration,

$$\nabla_{\boldsymbol{\phi}} \mathcal{L}(\boldsymbol{\phi}) \approx \sum_{t=1}^{T} \nabla_{\boldsymbol{\phi}}\left[\log p\left(\mathbf{X}, \boldsymbol{\theta}_t, \boldsymbol{\alpha}_t^{-1}, \beta_t\right) - \log q\left(\boldsymbol{\theta}_t, \boldsymbol{\alpha}_t^{-1}, \beta_t; \boldsymbol{\phi}\right)\right]$$

where T corresponds to the number of samples we draw from $q\left(\boldsymbol{\theta}, \boldsymbol{\alpha}^{-1}, \beta; \phi\right)$ during a single training step (by default $T = 1$).

We implemented scPCA using the pyro probabilistic programming language. By default we train the models for 30000 epochs, employ an ADAMGrad optimizer with a multi-step learning rate decay and an initial learning rate of 0.1. For scalability, we compute stochastic gradients over batches of observations.

Relationship to other factor model approaches

In the following section we compare scPCA to other factor models and related modeling approaches which incorporate secondary variables into their analyses.

Principal component analysis (PCA)

PCA projects high-dimensional data into a lower-dimensional space, maximizing the variance explained by the projection. Consider a mean-centered data matrix $\mathbf{X} \in \mathbb{R}^{C \times G}$ for C observations and G features. To perform a PCA on $\mathbf{X}$ we may employ a singular value decomposition (SVD)

$$\mathbf{X} = \mathbf{U}\mathbf{S}\mathbf{V}^T \text{ where } \mathbf{U}^T\mathbf{U} = \mathbf{V}^T\mathbf{V} = \mathbf{I}$$

where $\mathbf{U}$ and $\mathbf{V}$ denote the left and right singular matrices, respectively, and $\mathbf{S}$ is a diagonal matrix containing the singular values. The decomposition reveals that $\mathbf{Z} = \mathbf{U}\mathbf{S}$ and $\mathbf{W} = \mathbf{V}^T$ such that

$$\mathbf{X} = \mathbf{Z}\mathbf{W}$$

where $\mathbf{Z}$ denotes the factor matrix and $\mathbf{W}$ the corresponding loading matrix. The linearity assumption ensures that the resulting loading vectors from PCA are simple and interpretable.

Probabilistic principal component analysis (PPCA)

The probabilistic formulation of PCA may be derived from a Gaussian latent variable model, which seeks to relate the G -dimensional observations $\mathbf{X} = \{\mathbf{x}_c\}$ to a F-dimensional ($F < G$) vector of latent variables through a linear model [4,5]. This relationship may be concisely denoted as

$$\mathbf{X} = \mathbf{Z}\mathbf{W} + \mathbf{E}$$

where $\mathbf{Z}$ a $C \times F$ factor matrix, $\mathbf{W}$ represents a $F \times G$-matrix loading matrix, and $\mathbf{E}$ a $C \times G$ residual matrix. Specifying an isotropic noise model induces a Gaussian on the marginal distribution of $\mathbf{x}$, i.e. $\mathbf{x} \sim \mathcal{N}(\boldsymbol{\mu}, \mathbf{W}\mathbf{W}^T + \sigma^2\mathbf{I})$, and it can be shown that as the covariance of the noise model becomes infinitesimal small $\sigma^2 \to 0$ standard PCA is recovered [4]. In this case, the F rows of $\mathbf{W}$ span a linear subspace that corresponds to the principal subspace of the data.

There are many variations and extensions of the PPCA model that enable it to handle binary[6] or categorical data[7]. For example, contrastive PCA[8,9] identifies components by contrasting the variance in a target dataset against a background dataset, thus highlighting unique patterns specific to the target. The GLM PCA framework[1] introduced an expectation-maximization algorithm to facilitate modeling count data with an over-dispersed negative binomial distribution.

The scPCA model is closely related to PPCA but enables to account for conditioning variables. Specifically, it assumes that the principal subspaces for each condition are closely related, allowing the bases for each condition to be expressed as a linear transformation of a reference basis/condition.

Principal component regression (PCR)

In principal component regression (PCR), we aim to predict a dependent variable $\mathbf{Y}$ using high-dimensional data $\mathbf{X}$[10,11]. This is straightforward when $\mathbf{X}$ is invertible, but it becomes challenging in high-dimensional settings where $\mathbf{X}$ is singular due to multicollinearity. PCR addresses this by performing PCA on $\mathbf{X}$ and then using the resulting factors $\mathbf{Z}$ as independent variables in a multiple regression on $\mathbf{Y}$. Since the PCA factors are uncorrelated, this approach mitigates the multicollinearity problem that often complicates high-dimensional analysis.

However, PCR does not inherently identify an optimal subset of predictors in $\mathbf{X}$. Instead, inspecting the prominent loading weights of the factors with the largest regression coefficients may hint at important features. Also note that the factors are chosen to optimally explain $\mathbf{X}$ rather than $\mathbf{Y}$, meaning PCR does not guarantee find factors that represent meaningful (i.e. predictive) covariates for $\mathbf{Y}$.

Partial least squares (PLS)

Partial least squares (PLS) finds factors from $\mathbf{X}$ that best predict $\mathbf{Y}$. Unlike PCR, which builds on factors explaining the variance in $\mathbf{X}$, PLS aims to find a basis for $\mathbf{X}$ that maximizes the variance explained in $\mathbf{Y}$. To achieve this, PLS finds factors $\mathbf{Z}$ that enable the simultaneous decomposition of $\mathbf{X}$ and $\mathbf{Y}$ such that the components explain as much as possible of the covariance between $\mathbf{X}$ and $\mathbf{Y}$. Specifically, we require that

$$\mathbf{X} = \mathbf{Z}\mathbf{W}^1 \text{ with } \mathbf{Z}^T\mathbf{Z} = \mathbf{I}$$

and

$$\hat{\mathbf{Y}} = \mathbf{Z}\mathbf{S}\mathbf{W}^2$$

where $\mathbf{W}^1$ represents a loading weight matrix for $\mathbf{X}$, $\mathbf{S}$ is a diagonal matrix with scaling factors, and $\mathbf{W}^2$ may be considered as a “weight matrix” of the dependent variable $\mathbf{Y}$[10,12]. Several algorithms have been proposed to perform PLS [13–15].

Canonical Correlation analysis (CCA)

Canonical Correlation Analysis (CCA) is a classical method for identifying correlations between two multivariate data sets, $\mathbf{X}^1 \in \mathbb{R}^{N \times D_1}$ and $\mathbf{X}^2 \in \mathbf{R}^{N \times D_2}$ [16–18] for N observations and D_m features ($m = \{1, 2\}$). Specifically, CCA finds two bases for the data such that the correlation between the projections of these variables onto these bases is maximized. In other words, CCA determines linear transformations for $\mathbf{X}^1$ and $\mathbf{X}^2$ so that the transformed coordinates are maximally correlated. CCA shares some similarities with PLS regression, with the key difference being that CCA treats both $\mathbf{X}^1$ and $\mathbf{X}^2$ symmetrically, while PLS uses one of the datasets to predict the other. Klami et al. proposed a Bayesian CCA method that incorporates sparse priors on the loading weights to enhance interpretability [19]. This model may be expressed

$$\mathbf{X}^1 = \mathbf{Z}\mathbf{W}^1 + \mathbf{E}^1 \text{ and } \mathbf{X}^2 = \mathbf{Z}\mathbf{W}^2 + \mathbf{E}^2$$

where $\mathbf{W}^1 \in \mathbb{R}^{F\times D_1}$ and $\mathbf{W}^2 \in \mathbb{R}^{F\times D_2}$ represent loading weight matrices comprising F factors, $\mathbf{Z} \in \mathbb{R}^{N\times F}$ a shared factor matrix and $\mathbf{E}^m \in \mathbb{R}^{N\times D_m}$ for $m = \{1, 2\}$ error matrices. Building on this, Multi-Omics Factor Analysis (MOFA)[20,21] generalized Bayesian CCA to accommodate multiple datasets. MOFA supports a range of likelihoods and incorporates sparsity priors, providing a more flexible and interpretable framework for analyzing complex data sets.

Sparse Contrastive PCA

Sparse contrastive PCA[22] is a deterministic, linear dimensionality reduction method designed to identify directions of variation that are enriched in a target dataset relative to a background dataset. It does so by optimizing a contrastive objective while enforcing sparsity in the loading vectors, thereby emphasizing features that distinguish the target from the background.

In contrast, scPCA takes a probabilistic, generative modeling approach. It learns a shared low-dimensional representation from reference (unperturbed) data and models how this basis is altered across experimental conditions through condition-specific linear transformations. Rather than directly contrasting target and background datasets, our scPCA explicitly captures shared variation and characterizes how each latent component shifts under different perturbations, providing a factor-wise view of biological variation across conditions.

CellANOVA

CellANOVA[23] is a post-hoc, projection-based method that builds on precomputed low-dimensional embeddings—typically derived from tools like Harmony[24] or Seurat[25]. It applies an ANOVA-style residual decomposition to partition variation into shared, batch-associated, and condition-specific factorisations.

By contrast, scPCA learns a shared low-dimensional basis and models condition effects as explicit linear transformations of this reference basis. This enables scPCA to provide interpretable, factor-specific perturbation readouts. Unlike CellANOVA, which attributes variation to residuals without an explicit link to latent factors, scPCA offers a mapping between latent axes and condition-induced changes. As a result, the two frameworks characterize condition effects through fundamentally different representational strategies.

Surrogate variable analysis (SVA)

SVA is a factor model based approach originally developed for DNA microarray chips with the aim to enable the unbiased detection of differential expressed genes with respect to some conditioning variables [26,27]. Specifically, the authors attribute gene expression changes to the presence of "primary variables" (in this manuscript "conditioning variable") and other unmodeled variables (expression heterogeneity (EH)), which may not be studied explicitly because measurements are not available, or it is simply inconvenient to do so. For example, one may be interested in quantifying changes in expression due to a disease condition. However, the expression of certain genes may also depend on the age, and some genes may be affected by both disease condition and age. In this example, disease condition may

be considered as a primary variable and age as a variable that contributes to expression heterogeneity. To account for EH the authors propose to

$$\mathbf{X} = \boldsymbol{\mu} + f(\mathbf{C}) + \mathbf{ZW} + \mathbf{E}$$

where μ represents a mean offset vector, $f(\mathbf{C})$ the effect of the primary variables, $\mathbf{ZW}$ models EH, and $\mathbf{E}$ a residual matrix. To estimate $\mathbf{ZW}$, the authors propose an iterative procedure in which they first subtract the signal due to the primary variables, and apply a low-rank decomposition to the resulting residual matrix. They then identify surrogate variables by pinpointing the subset of genes driving the signature of each significant EH component. Finally, the authors employ the surrogate variables as covariates for a regression analysis, thereby allowing to account for EH in the analysis.

In summary, while scPCA, (P)PCA and CCA reconstruct linear hyperplanes which encompass both primary and unmodelled variables, SVA explicitly tries to reconstruct a subspace that mainly explains expression heterogeneity. In this way, SVA enables an unbiased assessment of the effects of the primary variable on gene expression.

Remove Unwanted Variation (RUV-2)

The RUV-2 model was originally developed for microarray data[28], and may be considered as an extension to SVA. The authors propose to decompose the gene expression matrix

$$\mathbf{X} = \mathbf{CW}^1 + \mathbf{ZW}^2 + \mathbf{E}$$

where $\mathbf{C}$ is a matrix whose columns encode the conditioning variable of interest, $\mathbf{Z}$ represents unobserved (hidden) factors representative for unwanted variation, and $\mathbf{W}^1$ and $\mathbf{W}^2$ matrices that harbor coefficients measuring the influence of the conditioning variable or factor on a gene (analogously to loading matrices). Note that we have dropped here an optional term for known observed covariates which are not of interest (e.g. batch).

A major difference between SVA and RUV-2 is in the way that both algorithms estimate the factors of unwanted variation. While SVA first subtracts the signal due to the conditioning variable from the data and decomposes the residuals to estimate the subspace of unwanted variation, RUV leverages negative control genes, i.e. a set of genes which are known to be unassociated with the conditioning variable. In other words, to estimate $\mathbf{Z}$, the RUV-2 algorithm first decomposes

$$\mathbf{X}_c = \mathbf{ZW}_c^2 + \mathbf{E}_c$$

where c denotes the set of negative control genes and $\mathbf{W}_c^2$ a loading matrix, and $\mathbf{E}_c$ a residual matrix. The estimate of $\mathbf{Z}$ is then plugged into equation above and $\mathbf{W}^1$ and $\mathbf{W}^2$ are estimated via regression. The original RUV-2 model has been adapted to RNA-seq data[29,30].

Gene Expression Decomposition and Integration (GEDI)

The GEDI factorisation framework[31] prescribes a specific parametrization to model the lower-dimensional subspaces of multi-condition single cell data. Particularly, the authors propose

$$\mathbf{X} = \boldsymbol{\mu}_r + \boldsymbol{\mu}_{i(c)} + (\mathbf{W}_r + \mathbf{W}_{i(c)})\mathbf{Z} + \mathbf{E}$$

where $\boldsymbol{\mu}_r$ represents the origin point for the reference plane (mean offset) spanned by the basis defined in $\mathbf{W}_r$, $\boldsymbol{\mu}_{i(c)}$ a sample-specific offset to the origin point of the reference ($i(c)$ denotes an indicator function for cell c) and $\mathbf{W}_{i(c)}$ a sample-specific transformation of the reference basis, $\mathbf{Z}$ a factor matrix, and $\mathbf{E}$ a residual matrix.

In scPCA, we generalize the GEDI model by introducing design (and/or indicator) matrices to flexibly parameterise the mean offset vector and the loading matrices. This allows scPCA to model arbitrary experimental designs including multiple conditioning variables as well as interaction effects. Additionally, we model the single-cell expression count matrix as negative binomial distributed thus mitigating the need to preprocess the data.

Supplementary Figures

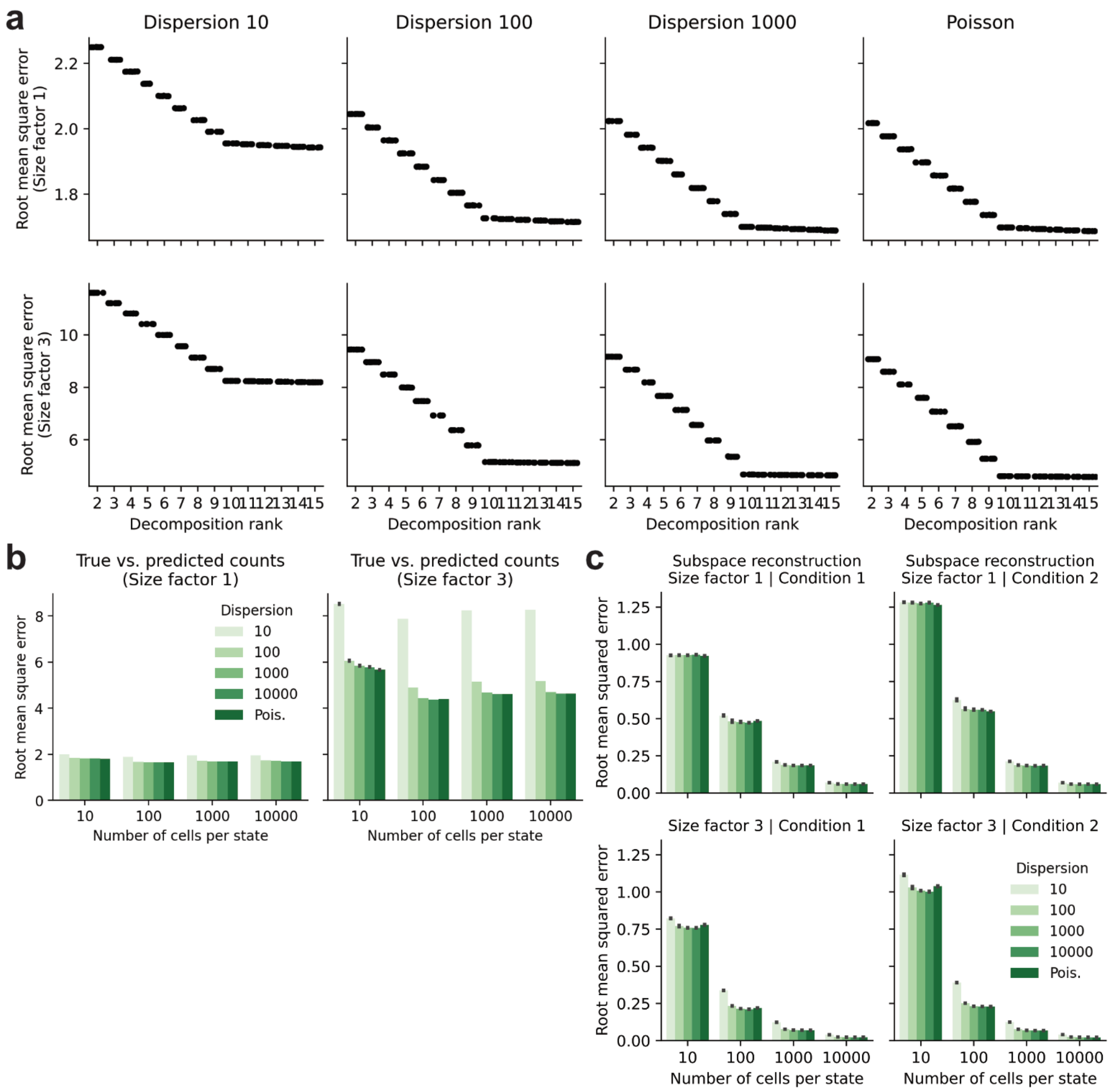

Supplementary Figure 1: Simulation results For **a., b.** and **c.** In-silico data comprising two conditions were simulated according to the generative process of the model (**Methods**) to assess how well the scPCA inference reconstructs simulated ground truth. The parameters of these simulations were chosen to cover a range of noise levels (overdispersion), average number of counts per cell (size factor) and number of cells per condition. **a.** Model selection via the root mean squared error (RMSE) of true and predicted counts. Data was simulated using ten basis vectors ($F = 10$), two conditions $Q = 2$ and $C_q = 1000$ cells per condition. For each parameter combination, we performed 10 scPCA decompositions for the ranks 2 to 15. **b.** RMSE of true and predicted counts for dataset of different sizes (total number of cells) and number of counts per cell (size factor). **c.** RMSE of the true and predicted subspace of the first and second simulated condition of the data.

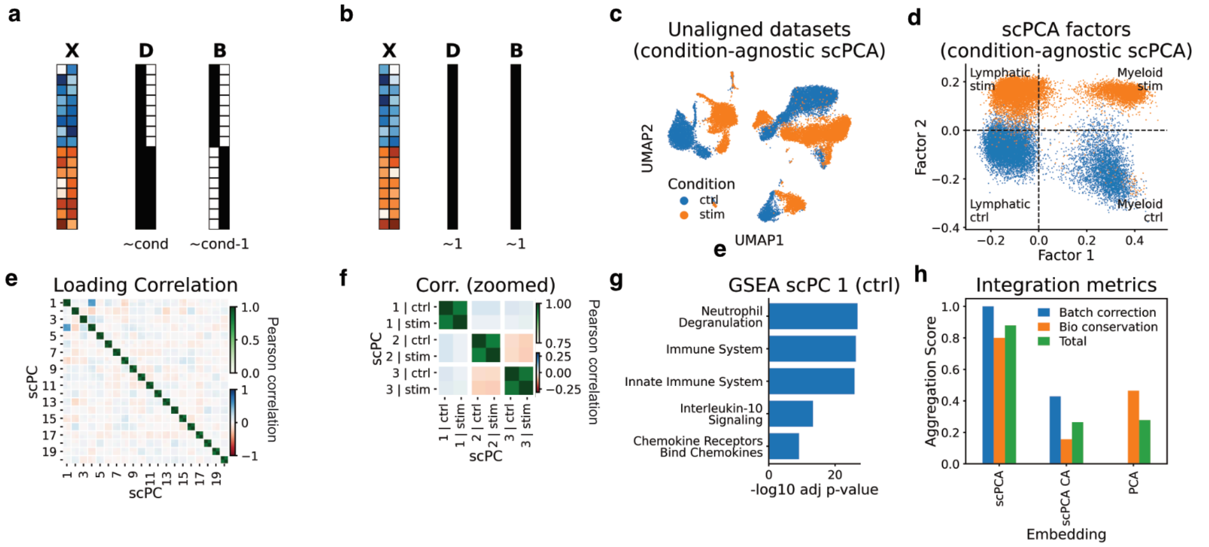

Supplementary Figure 2: Modeling untreated and IFN beta stimulated PBMCs. a. and b. Schematic depiction of the input data for the condition-informed (**a**) and condition-agnostic (**b**) scPCA factorizations. These include the expression count matrix **X** with cells from the control (blue) and stimulated (orange) condition, alongside the design and indicator matrices **D** and **B**. The design matrix **D** uses the control condition as a reference (column of ones) and models the IFN-beta condition as a deviation from this baseline (**a**). The indicator matrix **B** is used to fit independent mean offsets for each condition (**a**). Conversely, using columns of ones as design matrices in scPCA results in dimensionality reduction that is agnostic of condition differences (**b**). **c.** UMAP plot based on the condition-agnostic scPCA factorization. **d.** Scatter plots of the first two scPCA factors (condition-agnostic). **e. and f.** The heatmap of loading weight cross correlations indicate low correlations between different components (red/blue colorbar), yet high correlations between the same component across conditions (green colorbar). **g.** Gene set enrichment analysis based on the top 200 loading weights of scPC1 (condition-informed). **h.** Comparison of batch integration and bio-conservations scores for cell embeddings generated by condition-aware and agnostic (CA) scPCA models as well as conventional PCA.

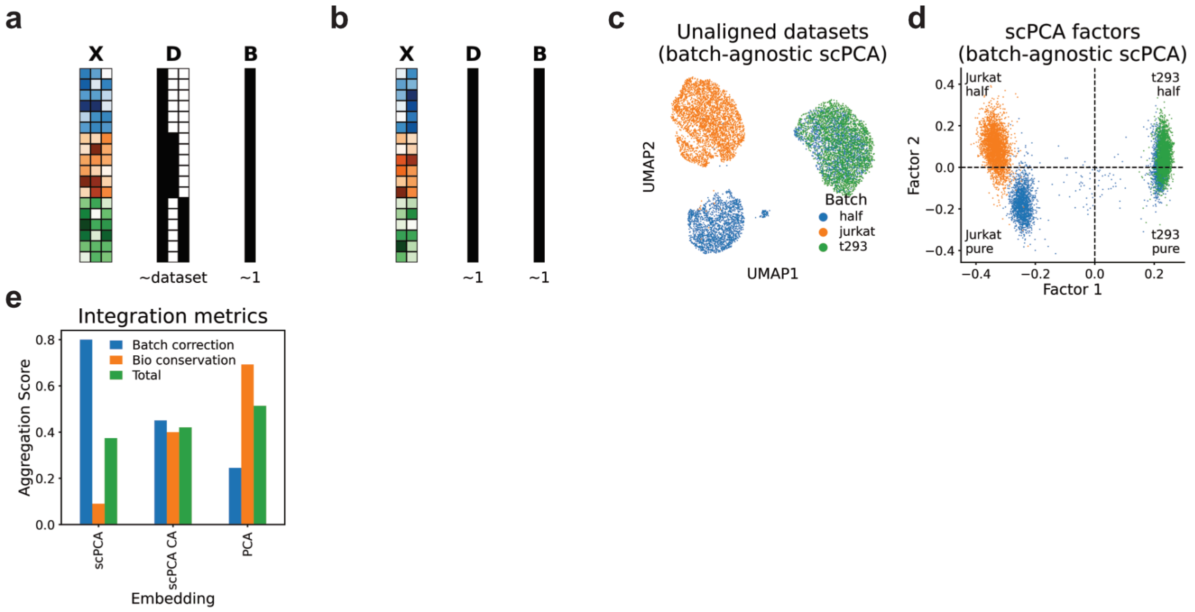

Supplementary Figure 3: a. and b. Schematic depiction of the input data for the batch-informed (**a**) and batch-agnostic (**b**) scPCA factorizations, including the expression count matrix **X** with cells from the half-half batch (blue), pure Jurkat batch (orange), and pure t293 batch (green), alongside the design and intercept matrices **D** and **B**. In (**a**), the loading design matrix **D** sets the half-half batch as a reference (column of ones) and aligns the components for the Jurkat and t293 batches, respectively. An indicator matrix of ones allows the model to fit a single mean offset across all batches. Conversely, using columns of ones for both design and indicator matrices leads to dimensionality reduction that ignores the batch structure in the data (**b**). **c.** The UMAP plot of Jurkat and t293 cells on the basis of the batch-agnostic scPCA factorisation results in a representation in which Jurkat cells separate by batch. **d.** Scatter plot of the first two factors from the batch-agnostic scPCA factorization, highlighting how factor 2 distinctly separates Jurkat cells by batch. **e.** Comparison of batch integration and bio-conservations scores for cell embeddings generated by the batch-aware and agnostic scPCA models as well as conventional PCA.

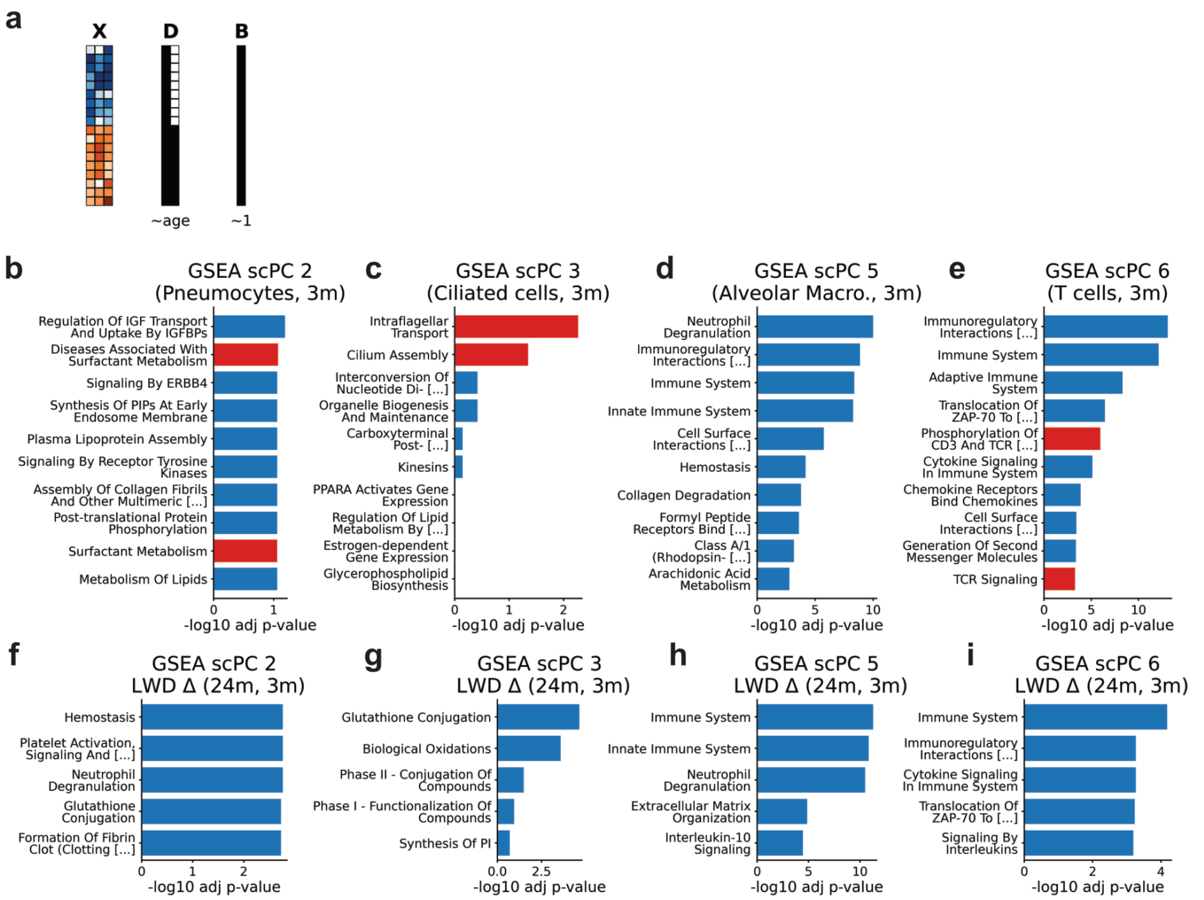

Supplementary Fig. 4: a. Schematic depiction of the input data including the expression count matrix **X** with lung cells from the 3 (blue) and 24 (orange) month old mice, alongside the design and intercept matrices **D** and **B**. **b.-e.** GSEA based on the top loading weights of indicated scPCs. Highlighted gene sets indicate particularly relevant programs (see main text). **f.-i.** GSEA based on positive LWDs of scPC2, 3, 5 and 6 indicate upregulated gene programs in aging mice.

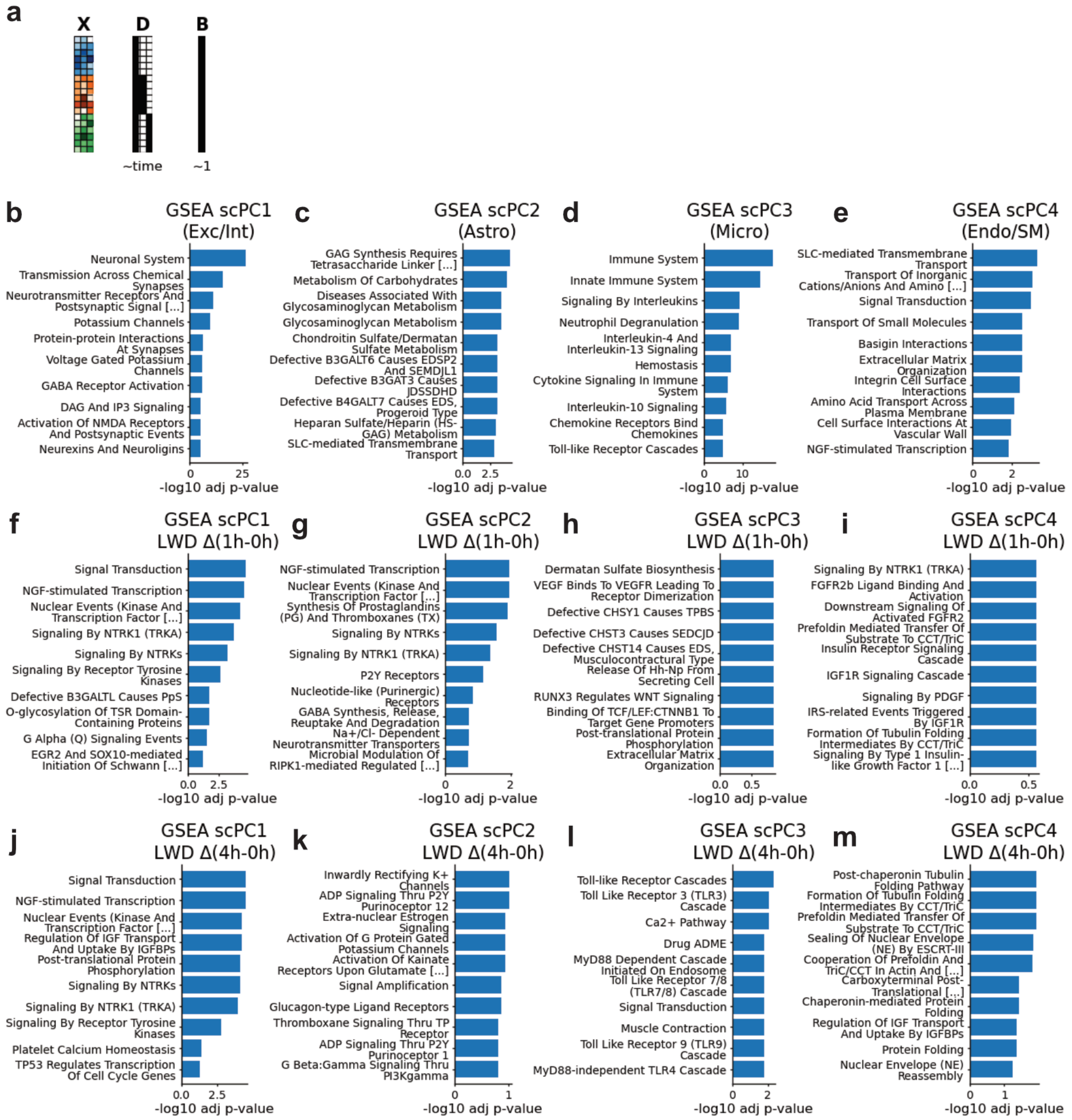

Supplementary Figure 5: a. Schematic depiction of the input data for scPCA including the expression count matrix **X** with mouse cortex cells from the timepoints 0 (blue), 1 (orange) and 3 hours, alongside the design and intercept matrices **D** and **B**. **b.-e.** GSEA based on the top loading weights of indicated scPCs. Highlighted gene sets indicate particularly relevant programs. **f.-i.** GSEA based on positive LWDs of scPC1-4 indicate upregulated gene programs across the 1h and 0h timepoints. **j.-m.** GSEA based on positive LWDs of scPC1-4 indicate upregulated gene programs across the 5h and 0h timepoints.

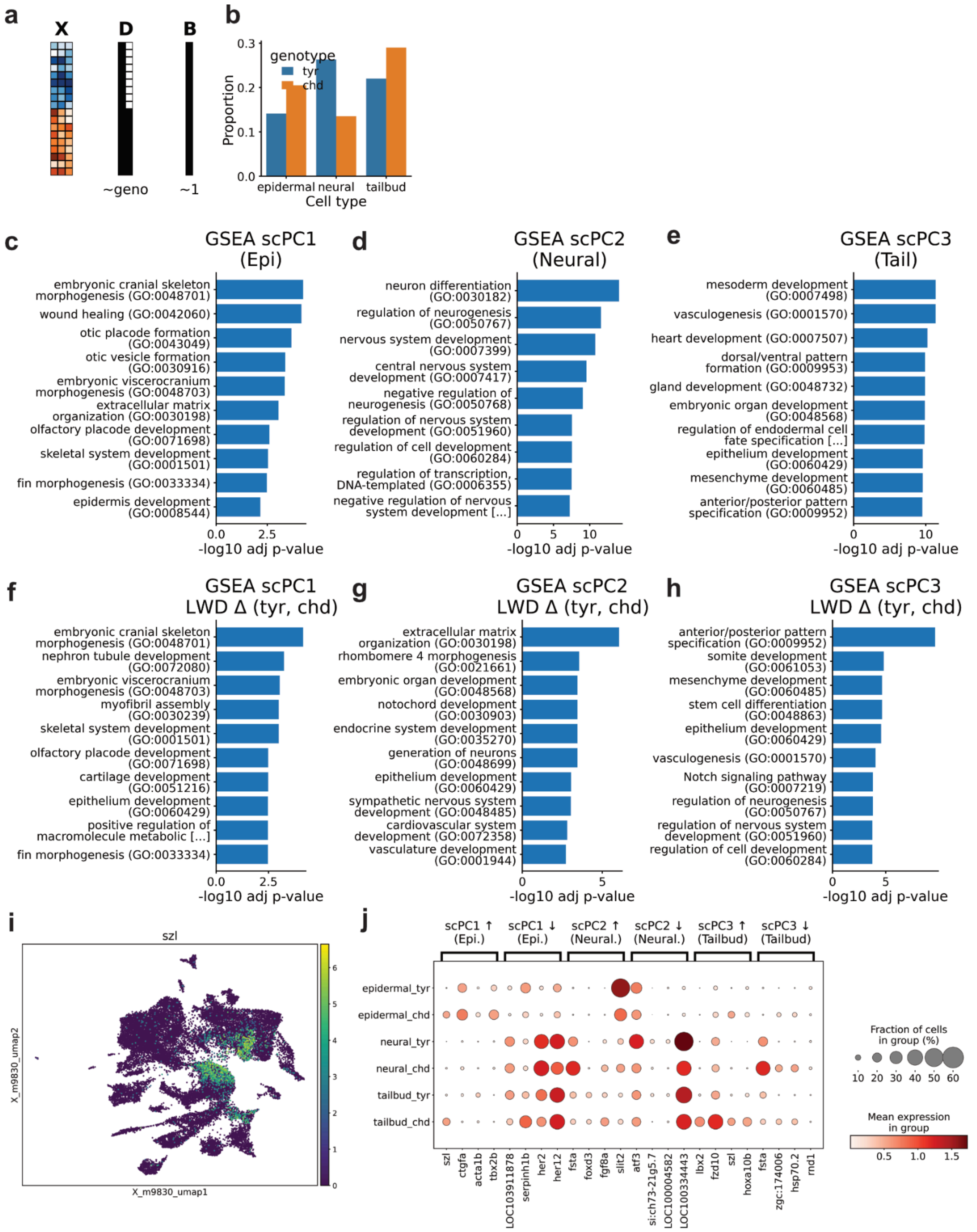

Supplementary Figure 6: a. Schematic depiction of the input data for scPCA including the expression count matrix **X** with zebrafish cells from the control (blue) and chordin knockout (orange), alongside the design and intercept matrices **D** and **B**. **c.-e.** GSEA based on the top loading weights of indicated scPCs. **f.-h.** GSEAs based on positive LWDs of scPC1-3. **i.** UMAP plot showing the expression of *szl* in distinct epidermal and tailbud cells. **j.** Dot plot representation of selected genes exhibiting the largest LWDs in indicated scPCs.

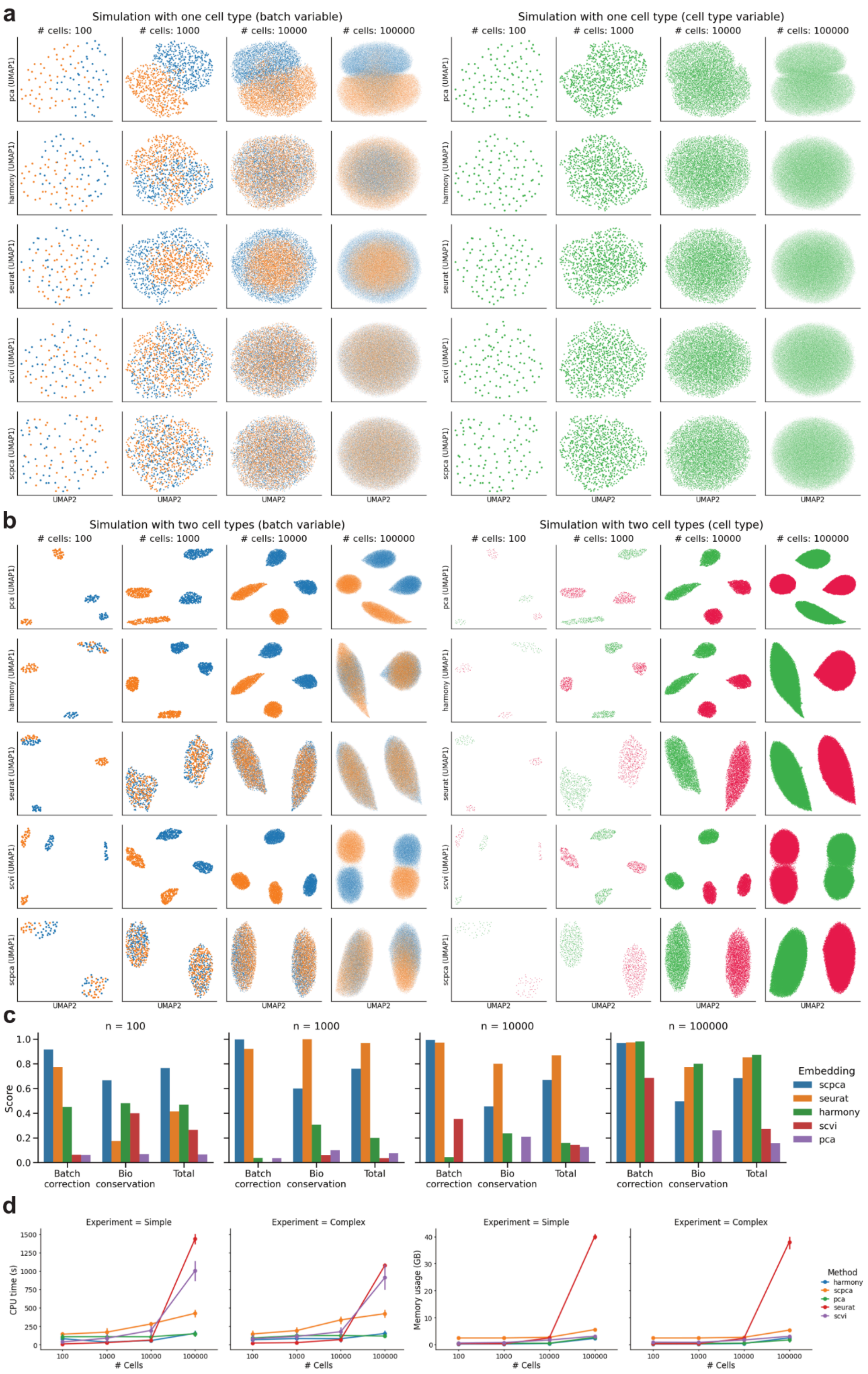
a
Simulation with one cell type (batch variable)
Simulation with one cell type (cell type variable)
cells: 100
cells: 1000
cells: 10000
cells: 100000
pca (UMAP1)
harmony (UMAP1)
seurat (UMAP1)
scvi (UMAP1)
scpca (UMAP1)
UMAP2
b
Simulation with two cell types (batch variable)
Simulation with two cell types (cell type)
c
n = 100
n = 1000
n = 10000
n = 100000
Score
Batch correction
Bio conservation
Total
Embedding
scpca
seurat
harmony
scvi
pca
d
Experiment = Simple
Experiment = Complex
CPU time (s)
Memory usage (GB)
Cells
Method
harmony
scpca
pca
seurat
scvi

Supplementary Figure 7: Benchmarking scPCA against PCA, Harmony, Seurat, and scVI on simulated datasets of varying complexity and size. a and b. UMAP visualizations of low-dimensional embeddings obtained using five integration methods (PCA, Harmony, Seurat (CCA), scVI, and scPCA) across two simulation scenarios with increasing dataset sizes (100, 1,000, 10,000, and 100,000 cells). Dots represent cells, colors either batch (blue/orange) or cell type (green/red). **a.** Single-cell simulations (**Methods**) with one cell type and **b.** Simulations involving two distinct cell types. **c.** Average biological conservation (Isolated labels, KMeans NMI, KMeans ARI, cLISI), batch correction (Silhouette batch, KBET, Graph connectivity, PCR comparison) and overall performance scores across the simulation experiments. **d.** Runtime and memory usage for each method as a function of dataset size, evaluated on both simple and complex simulation scenarios.

Extended Data Figures

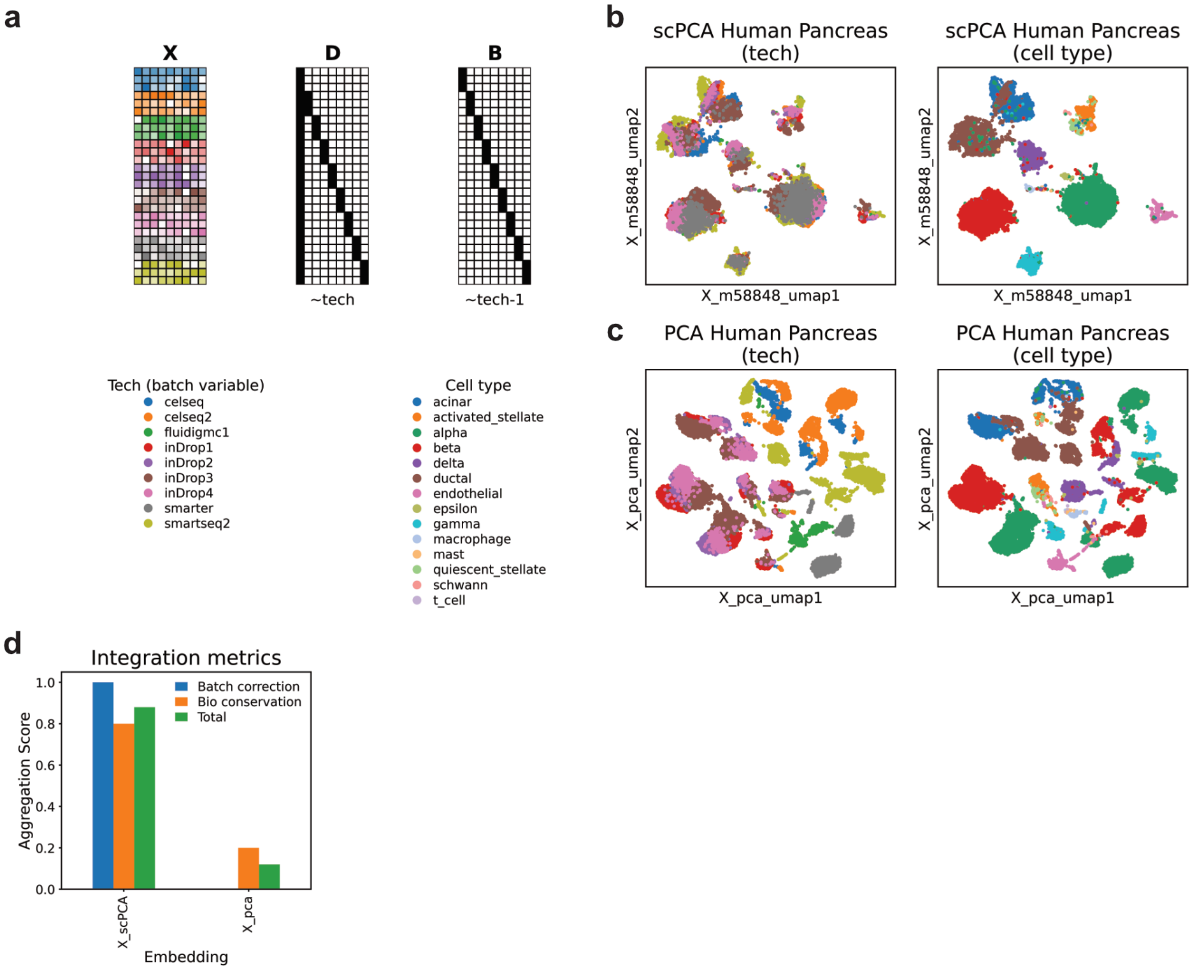

Extended Data Figure 1: Integrating the human pancreas single-cell dataset with scPCA. a. Schematic depiction of the human pancreas dataset **X** comprising single-cell data from 9 different sequencing platforms, along with the corresponding design and indicator matrices **D** and **B**, which reflect the batch structure in the data. **b.** UMAP plots based on scPCA cell embeddings showing annotations for the batch variable (tech) and cell types. **c.** UMAP plots based on PCA cell embeddings showing annotations for the batch variable and cell types. **d.** Bio conservation and batch integration scores for PCA and scPCA embeddings.

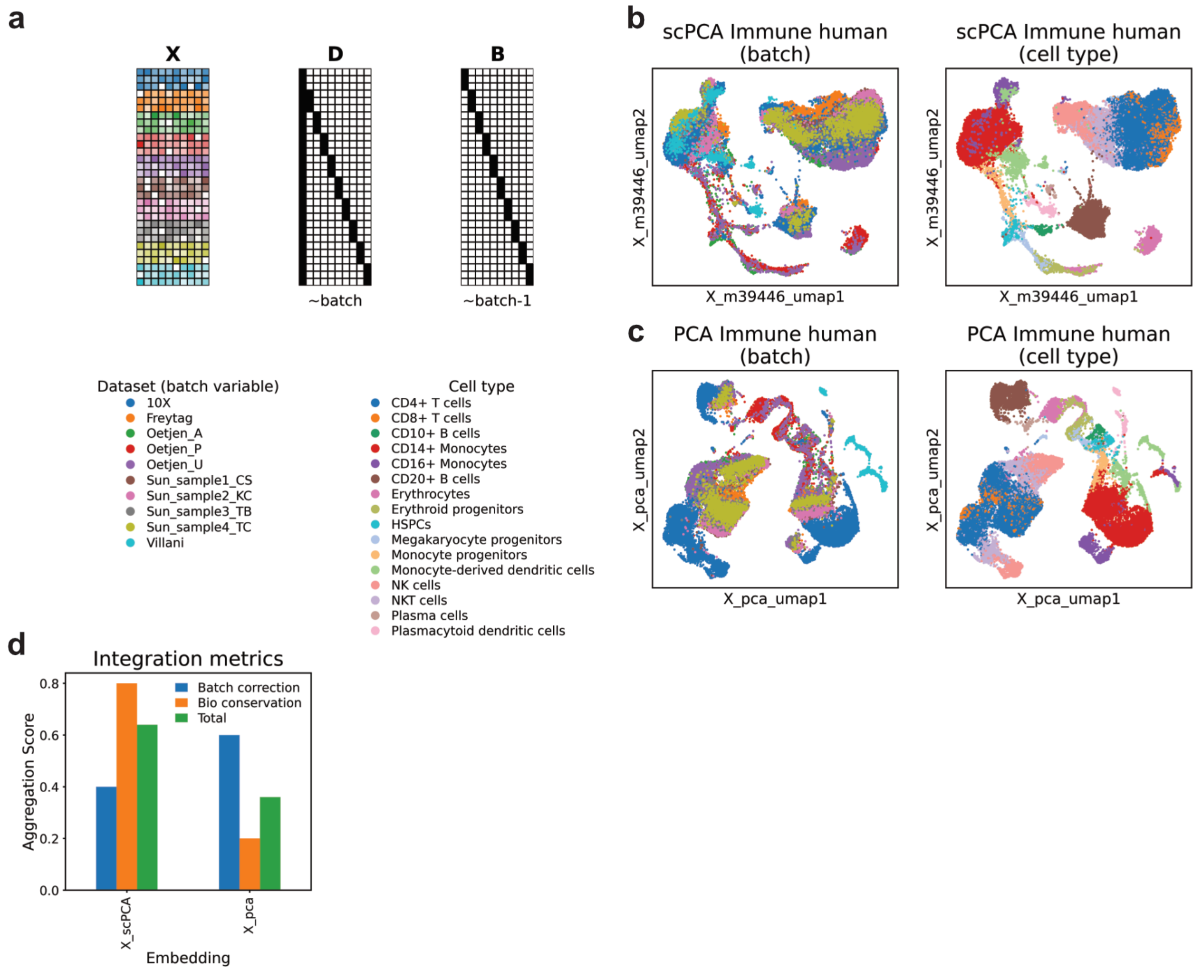

Extended Data Figure 2: Integrating the human immune dataset with scPCA. a. Schematic depiction of the human immune dataset **X** comprising single-cell data from 10 donors, along with the corresponding design and indicator matrices **D** and **B**, which reflect the donor structure in the data. **b.** UMAP plots based on scPCA cell embeddings showing annotations for the batch variable and cell types. **c.** UMAP plots based on PCA cell embeddings showing annotations for the batch variable and cell types. **d.** Bio conservation and batch integration scores for PCA and scPCA embeddings.

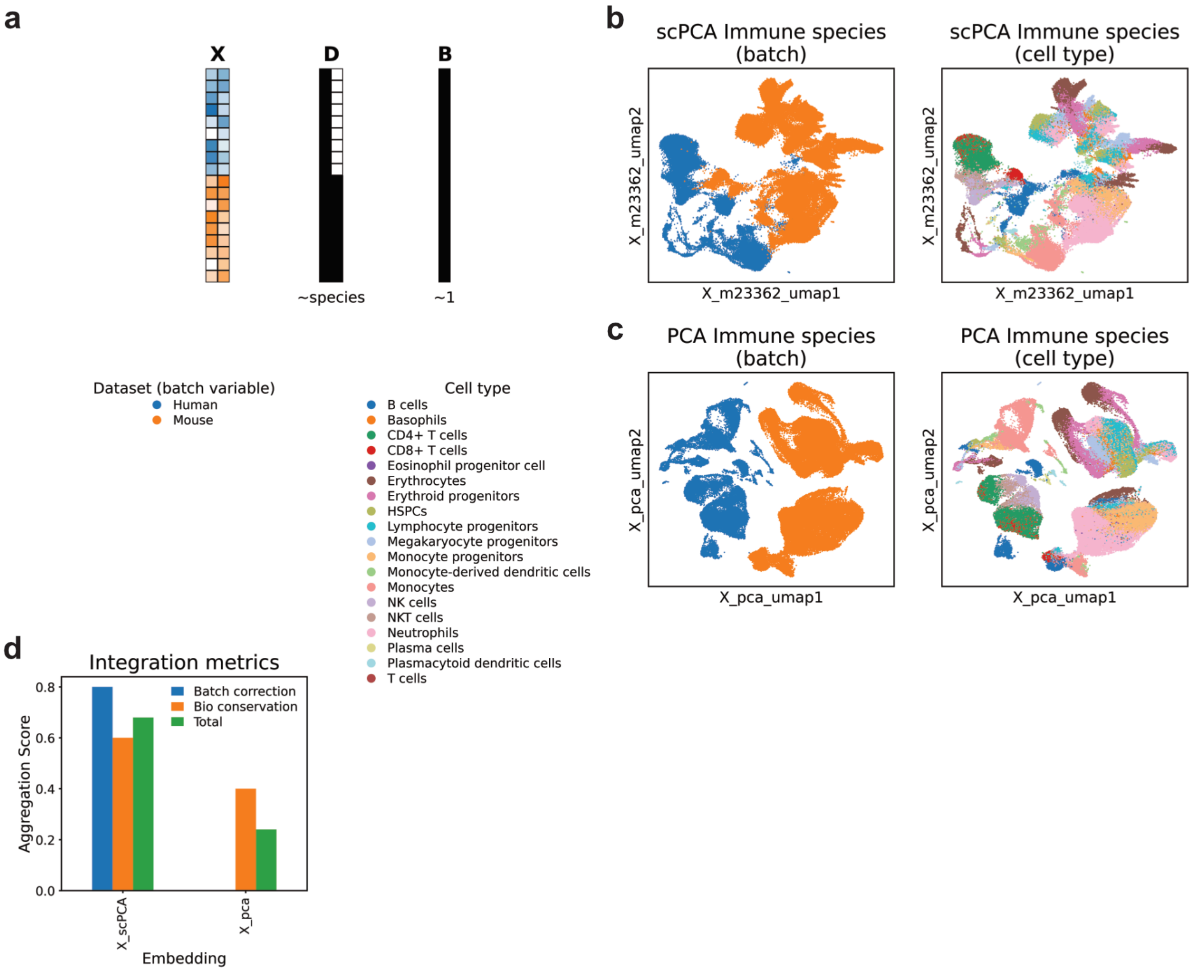

Extended Data Figure 3: Integrating immune cells across species using scPCA. a. Schematic depiction of the mouse immune dataset **X** comprising single-cell data from 2 species, along with corresponding design and indicator matrices **D** and **B**, which reflect the batch structure in the data. **b.** UMAP plots based on scPCA cell embeddings showing annotations for the batch variable and cell types. **c.** UMAP plots based on PCA cell embeddings showing annotations for the batch variable and cell types. **d.** Bio conservation and batch integration scores for PCA and scPCA embeddings.

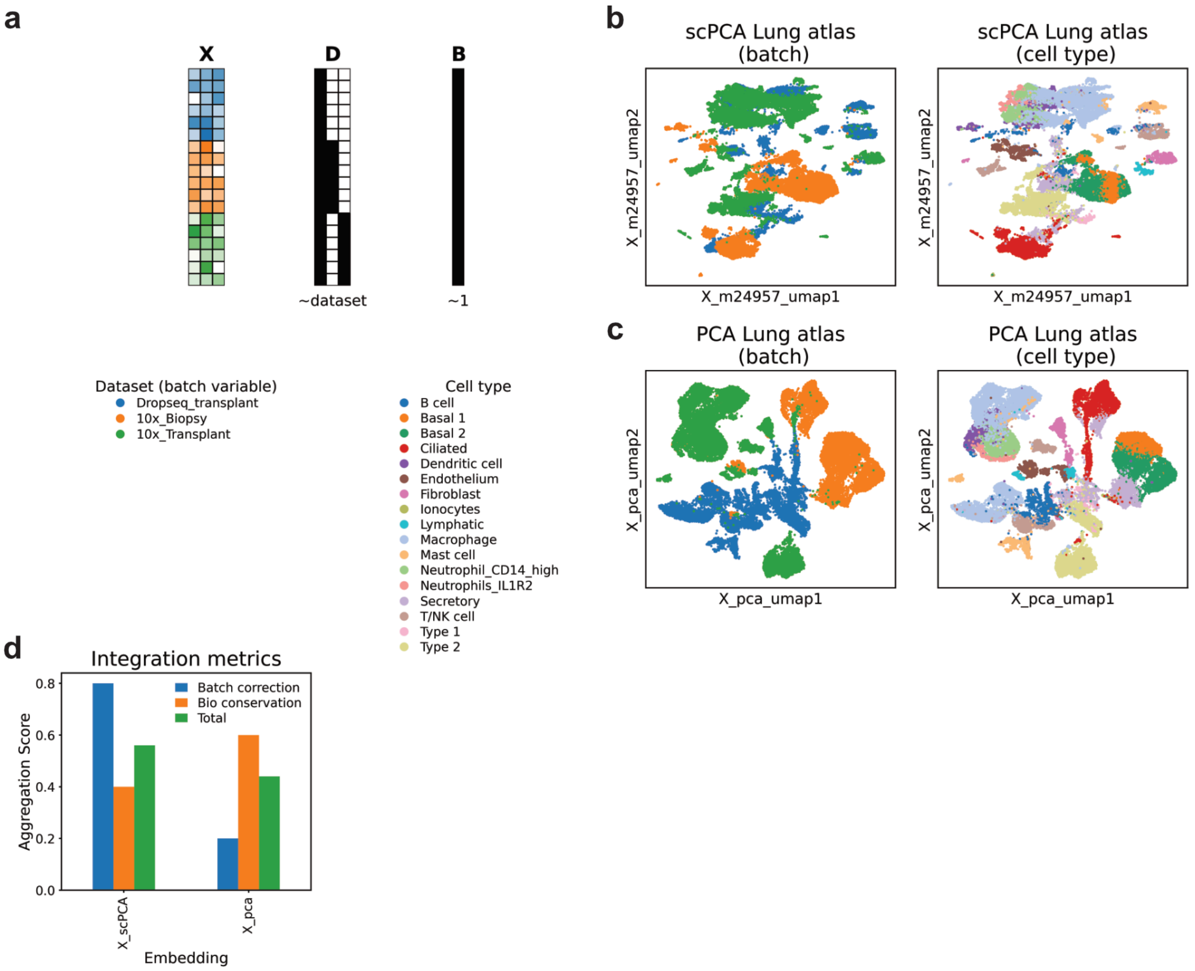

Extended Data Figure 4: Integrating the lung dataset across protocols with scPCA. a. Schematic depiction of the lung dataset **X**, and corresponding design and indicator matrices **D** and **B**, which reflect the batch structure in the data. **b.** UMAP plots based on scPCA cell embeddings showing annotations for the batch variable and cell types. **c.** UMAP plots based on PCA cell embeddings showing annotations for the batch variable and cell types. **d.** Bio conservation and batch integration scores for PCA and scPCA embeddings.

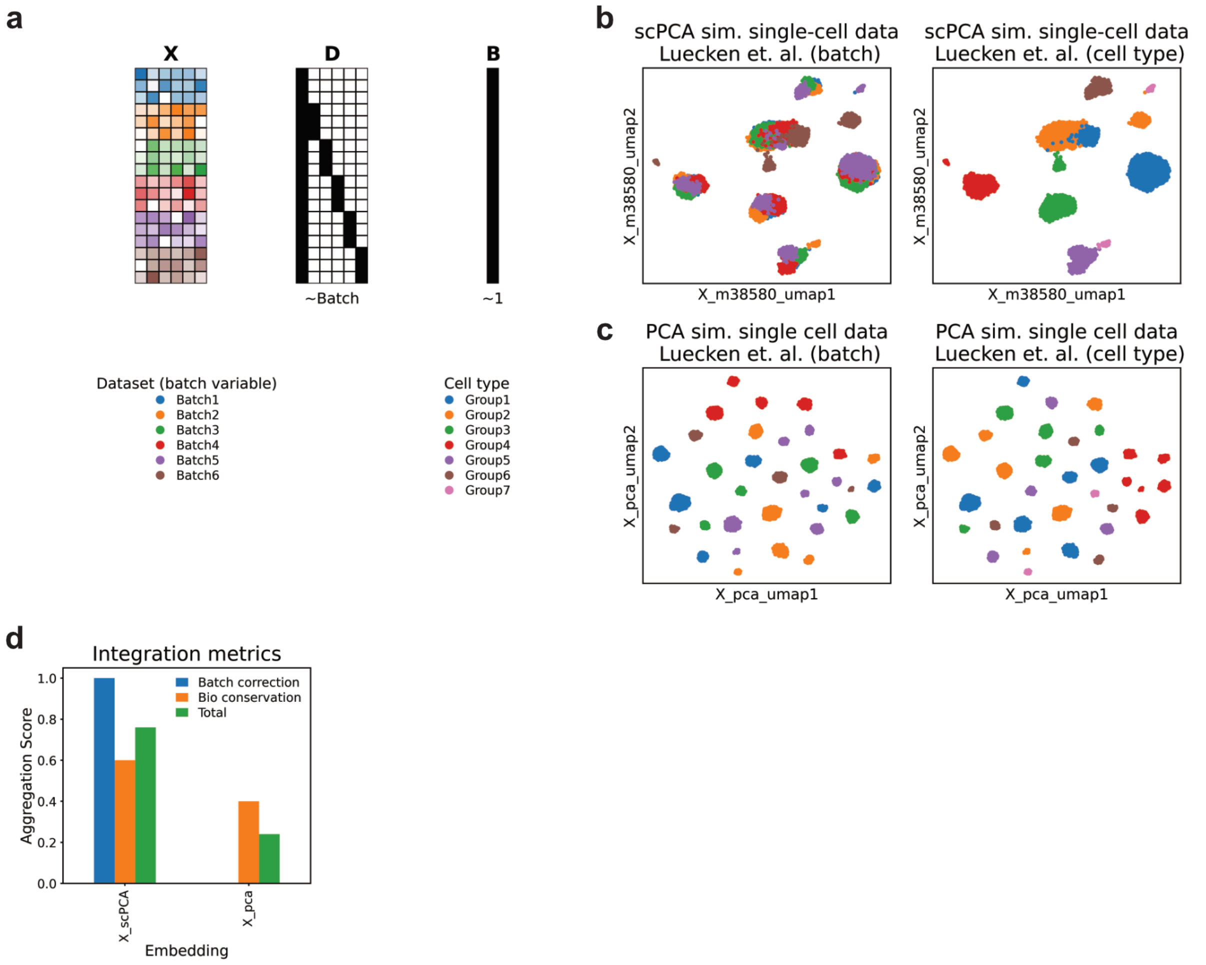

Extended Data Figure 5: Integrating simulated single-cell data with scPCA. a. Schematic depiction of the simulated single-cell dataset by Luecken et al., and corresponding design and indicator matrices **D** and **B**, which reflect the batch structure in the data. **b.** UMAP plots based on scPCA cell embeddings showing annotations for the batch variable and cell types. **c.** UMAP plots based on PCA cell embeddings showing annotations for the batch variable and cell types. **d.** Bio conservation and batch integration scores for PCA and scPCA embeddings.

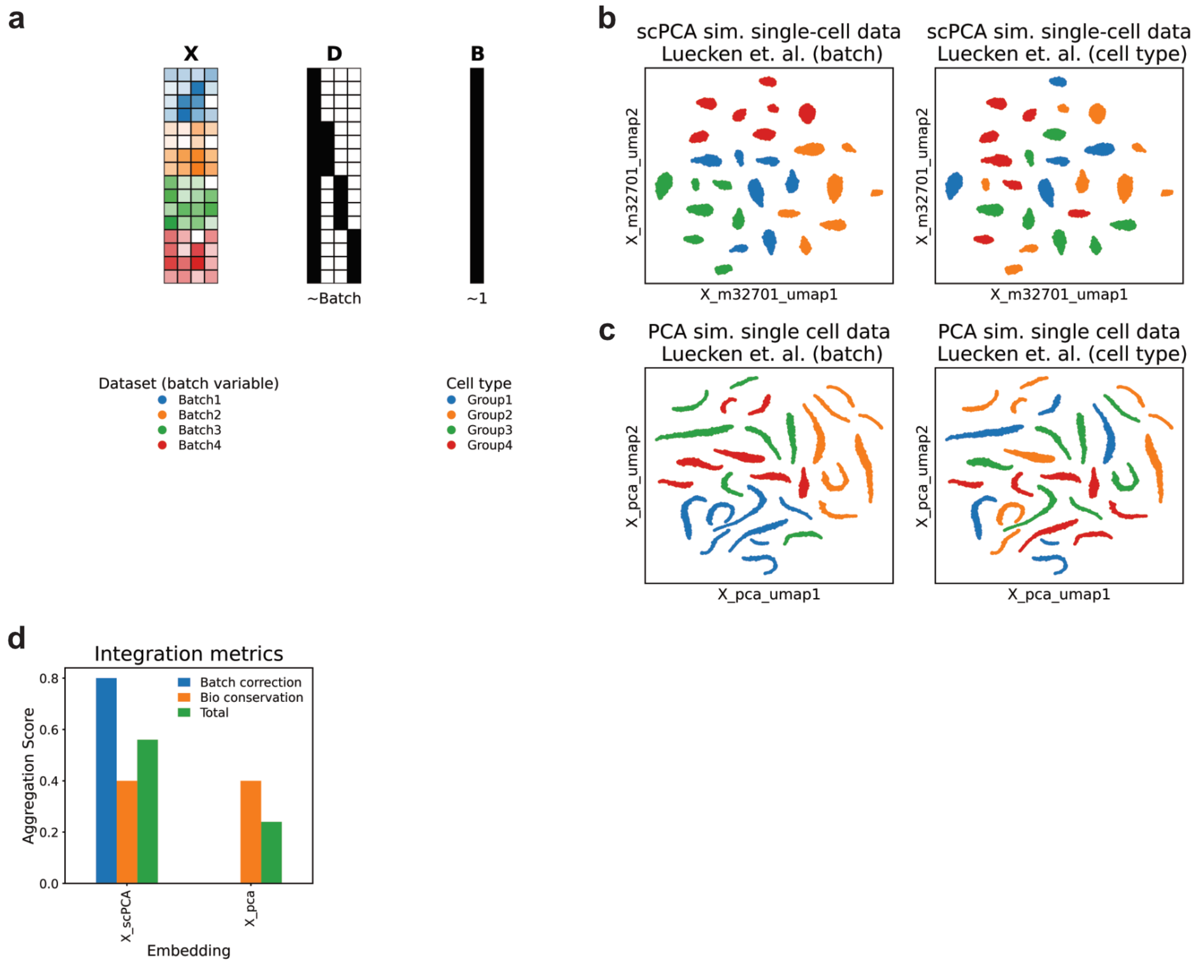

Extended Data Figure 6: Integrating complex simulated single-cell data with scPCA. a. Schematic depiction of Luecken et al. second simulated single-cell dataset, and corresponding design and indicator matrices **D** and **B**, which reflect the batch structure in the data. **b.** UMAP plots based on scPCA cell embeddings showing annotations for the batch variable (tech) and cell types. **c.** UMAP plots based on PCA cell embeddings showing annotations for the batch variable and cell types. **d.** Bio conservation and batch integration scores for PCA and scPCA embeddings.

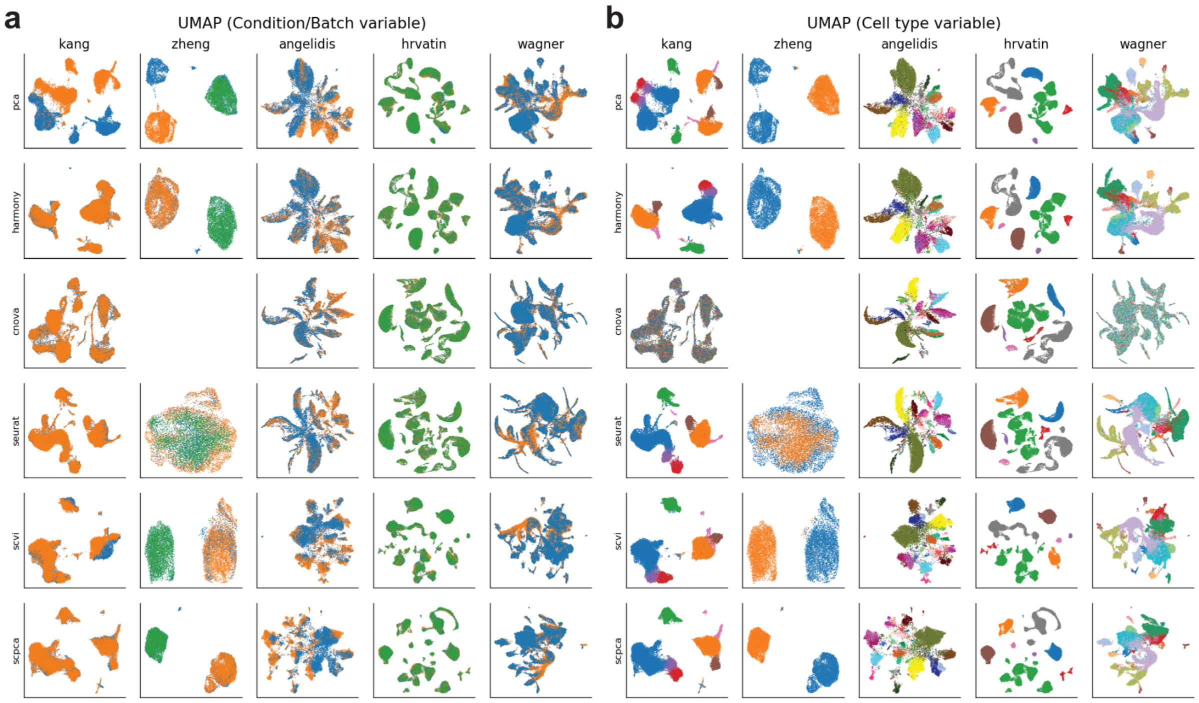

Extended Data Figure 7: Comparison of scPCA embeddings with other integration methods on published single-cell datasets. **a. and b** UMAP visualizations of cell embeddings generated by PCA, Harmony (harmony), CellANOVA (cnova), Seurat (seurat), and scVI (scvi) across datasets from Kang et al. (kang), Zheng et al. (zheng), Hrvatin et al. (hrvatin), and Wagner et al. (wagner) colored by condition variable (**a**) and cell types (**b**).

a Kang et al.

	Bio conservation					Batch correction					Aggregate score		
**Method**	Isolated labels	KMeans NMI	KMeans ARI	Silhouette label	cLISI	Silhouette batch	iLISI	KBET	Graph connectivity	PCR comparison	Batch correction	Bio conservation	Total
**X_seurat**	1.00	1.00	1.00	1.00	1.00	0.89	0.42	0.96	0.98	0.99	0.85	1.00	0.94
**X_scpca**	0.75	0.91	0.74	0.55	1.00	0.97	0.93	1.00	0.95	1.00	0.97	0.79	0.86
**X_harmony**	0.64	0.88	0.68	0.73	1.00	0.92	0.78	0.93	0.90	0.98	0.90	0.78	0.83
**X_scvi**	0.49	0.64	0.44	0.36	1.00	1.00	0.40	0.50	1.00	0.75	0.73	0.58	0.64
**X_pca**	0.51	0.69	0.42	0.47	1.00	0.00	0.00	0.00	0.91	0.00	0.18	0.62	0.44
**X_cnova**	0.00	0.00	0.00	0.00	0.00	0.87	1.00	0.77	0.00	0.96	0.72	0.00	0.29

b Zheng et al.

	Bio conservation					Batch correction					Aggregate score		
**Method**	Isolated labels	KMeans NMI	KMeans ARI	Silhouette label	cLISI	Silhouette batch	iLISI	KBET	Graph connectivity	PCR comparison	Batch correction	Bio conservation	Total
**X_harmony**	1.00	1.00	1.00	1.00	1.00	0.92	0.71	1.00	0.80	0.00	0.69	1.00	0.87
**X_scpca**	0.45	0.96	0.98	0.45	1.00	0.94	0.64	0.71	0.00	0.51	0.56	0.77	0.68
**X_scvi**	0.17	0.06	0.07	0.17	1.00	1.00	0.64	0.63	0.77	0.83	0.77	0.30	0.49
**X_pca**	0.96	0.01	0.00	0.96	1.00	0.00	0.00	0.00	1.00	0.00	0.20	0.59	0.43
**X_seurat**	0.00	0.00	0.00	0.00	0.00	0.44	1.00	0.47	0.05	1.00	0.59	0.00	0.24

c Angelidis et al.

	Bio conservation					Batch correction					Aggregate score		
**Method**	Isolated labels	KMeans NMI	KMeans ARI	Silhouette label	cLISI	Silhouette batch	iLISI	KBET	Graph connectivity	PCR comparison	Batch correction	Bio conservation	Total
**X_seurat**	0.76	1.00	1.00	1.00	1.00	0.43	1.00	0.50	0.47	1.00	0.68	0.95	0.84
**X_scpca**	0.77	0.83	0.38	0.64	0.90	0.72	0.00	0.00	0.88	0.00	0.32	0.70	0.55
**X_cnova**	1.00	0.27	0.61	0.42	0.27	0.00	0.89	1.00	0.00	0.97	0.57	0.52	0.54
**X_scvi**	0.52	0.06	0.08	0.00	0.39	1.00	0.66	0.38	1.00	0.54	0.72	0.21	0.41
**X_harmony**	0.00	0.00	0.00	0.35	0.00	1.00	0.70	0.86	0.36	0.72	0.73	0.07	0.33
**X_pca**	0.01	0.07	0.15	0.34	0.15	0.66	0.15	0.11	0.47	0.00	0.28	0.15	0.20

d Hrvatin et al.

	Bio conservation					Batch correction					Aggregate score		
**Method**	Isolated labels	KMeans NMI	KMeans ARI	Silhouette label	cLISI	Silhouette batch	iLISI	KBET	Graph connectivity	PCR comparison	Batch correction	Bio conservation	Total
**X_harmony**	0.48	0.96	0.96	0.92	1.00	1.00	0.96	1.00	0.93	0.85	0.95	0.86	0.90
**X_seurat**	1.00	1.00	1.00	1.00	1.00	0.35	0.59	0.27	0.85	0.86	0.59	1.00	0.83
**X_pca**	0.47	0.85	0.72	0.91	1.00	0.81	0.83	0.70	0.95	0.00	0.66	0.79	0.74
**X_scpca**	0.29	0.88	0.80	0.35	1.00	0.75	0.00	0.00	0.86	1.00	0.52	0.67	0.61
**X_scvi**	0.00	0.63	0.39	0.00	1.00	0.83	0.71	0.56	1.00	0.00	0.62	0.41	0.49
**X_cnova**	0.55	0.00	0.00	0.29	0.00	0.00	1.00	0.83	0.00	0.00	0.37	0.17	0.25

e Wagner et al.

	Bio conservation					Batch correction					Aggregate score		
**Method**	Isolated labels	KMeans NMI	KMeans ARI	Silhouette label	cLISI	Silhouette batch	iLISI	KBET	Graph connectivity	PCR comparison	Batch correction	Bio conservation	Total
**X_scvi**	0.89	0.84	0.74	0.97	1.00	0.94	0.31	0.89	1.00	0.52	0.73	0.89	0.82
**X_scpca**	0.98	0.91	0.80	1.00	1.00	0.72	0.00	0.77	0.97	0.32	0.56	0.94	0.79
**X_harmony**	0.62	0.91	0.77	0.76	0.97	0.74	0.52	1.00	0.97	0.38	0.72	0.81	0.77
**X_seurat**	1.00	1.00	1.00	0.50	1.00	0.00	0.38	0.91	0.95	0.57	0.56	0.90	0.76
**X_pca**	0.61	0.91	0.83	0.75	0.97	0.61	0.31	0.74	0.98	0.00	0.53	0.81	0.70
**X_cnova**	0.00	0.00	0.00	0.00	0.00	1.00	1.00	0.00	0.00	1.00	0.60	0.00	0.24

Extended Data Figure 8: Benchmarking of scPCA and alternative integration methods on published single-cell datasets using scIB metrics. a-e. Comparison of biological conservation and batch correction performance across published datasets from Kang et al. (**a**), Zheng et al. (**b**), Angelidis et al. (**c**), Hrvatin et al. (**d**), and Wagner et al. (**e**). Methods compared include scPCA (X_scpca), Seurat (X_seurat), Harmony (X_harmony), scVI (X_scvi), PCA (X_pca), and CellANOVA (X_cnova).

References

1. Townes, F. W., Hicks, S. C., Aryee, M. J. & Irizarry, R. A. Feature selection and dimension reduction for single-cell RNA-Seq based on a multinomial model. Genome Biol. 20, 295 (2019).
2. Blei, D. M., Kucukelbir, A. & McAuliffe, J. D. Variational Inference: A Review for Statisticians. J. Am. Stat. Assoc. 112, 859–877 (2017).
3. Tipping, M. E. & Bishop, C. M. Probabilistic principal component analysis. J. R. Stat. Soc. Series B Stat. Methodol. 61, 611–622 (1999).
4. Bishop, C. Bayesian pca. Adv. Neural Inf. Process. Syst. 11, (1998).
5. Tipping, M. E. Probabilistic visualisation of high-dimensional binary data. Adv. Neural Inf. Process. Syst. 592–598 (1998).
6. Khan, M. E., Marlin, B. M., Bouchard, G. & Murphy, K. P. Variational bounds for mixed-data factor analysis. Adv. Neural Inf. Process. Syst. 1108–1116 (2010).
7. Abid, A., Zhang, M. J., Bagaria, V. K. & Zou, J. Contrastive Principal Component Analysis. arXiv [stat.ML] (2017).
8. Li, D., Jones, A. & Engelhardt, B. Probabilistic Contrastive Principal Component Analysis. arXiv [stat.ME] (2020).
9. Abdi, H. Partial Least Squares (PLS) Regression. (2003).
10. Jolliffe, I. T. A Note on the Use of Principal Components in Regression. J. R. Stat. Soc. Ser. C Appl. Stat. 31, 300–303 (1982).
11. Abdi, H. Partial least squares regression and projection on latent structure regression (PLS Regression). Wiley Interdiscip. Rev. Comput. Stat. 2, 97–106 (2010).

12. Lindgren, F. & Rännar, S. Alternative Partial Least-Squares (PLS) Algorithms. in 3D QSAR in Drug Design: Recent Advances (eds. Kubinyi, H., Folkers, G. & Martin, Y. C.) 105–113 (Springer Netherlands, Dordrecht, 1998).
13. Westland, J. C. Partial Least Squares Path Analysis. in Structural Equation Models: From Paths to Networks (ed. Westland, J. C.) 17–38 (Springer International Publishing, Cham, 2019).
14. Sæbø, S., Almøy, T., Flatberg, A., Aastveit, A. H. & Martens, H. LPLS-regression: a method for prediction and classification under the influence of background information on predictor variables. Chemometrics Intellig. Lab. Syst. 91, 121–132 (2008).
15. Hardoon, D. R., Szedmak, S. & Shawe-Taylor, J. Canonical correlation analysis: an overview with application to learning methods. Neural Comput. 16, 2639–2664 (2004).
16. Hotelling, H. RELATIONS BETWEEN TWO SETS OF VARIATES. Biometrika 28, 321–377 (1936).
17. Abdi, H., Guillemot, V., Eslami, A. & Beaton, D. Canonical Correlation Analysis. in Encyclopedia of Social Network Analysis and Mining (eds. Alhajj, R. & Rokne, J.) 1–16 (Springer New York, New York, NY, 2017).
18. Klami, A., Virtanen, S. & Kaski, S. Bayesian Canonical correlation analysis. J. Mach. Learn. Res. 14, 965–1003 (2013).
19. Argelaguet, R. et al. Multi‐Omics Factor Analysis—a framework for unsupervised integration of multi‐omics data sets. Mol. Syst. Biol. 14, e8124 (2018).
20. Argelaguet, R. et al. MOFA+: a statistical framework for comprehensive integration of multi-modal single-cell data. Genome Biol. 21, 111 (2020).
21. Leek, J. T., Johnson, W. E., Parker, H. S., Jaffe, A. E. & Storey, J. D. The sva package for removing batch effects and other unwanted variation in high-throughput experiments. Bioinformatics 28, 882–883 (2012).
22. Leek, J. T. & Storey, J. D. Capturing heterogeneity in gene expression studies by surrogate variable analysis. PLoS Genet. 3, 1724–1735 (2007).
23. Gagnon-Bartsch, J. A. & Speed, T. P. Using control genes to correct for unwanted

variation in microarray data. Biostatistics 13, 539–552 (2012).

24. Risso, D., Ngai, J., Speed, T. P. & Dudoit, S. Normalization of RNA-seq data using factor analysis of control genes or samples. Nat. Biotechnol. 32, 896–902 (2014).
25. Molania, R. et al. Removing unwanted variation from large-scale RNA sequencing data with PRPS. Nat. Biotechnol. 41, 82–95 (2023).
26. Madrigal, A., Lu, T., Soto, L. M. & Najafabadi, H. S. A unified model for interpretable latent embedding of multi-sample, multi-condition single-cell data. bioRxiv 2023.08.15.553327 (2023) doi:10.1101/2023.08.15.553327.

1. Townes, F. W., Hicks, S. C., Aryee, M. J. & Irizarry, R. A. Feature selection and dimension reduction for single-cell RNA-Seq based on a multinomial model. *Genome Biol.* **20**, 295 (2019).
2. Svensson, V. Droplet scRNA-seq is not zero-inflated. *Nat. Biotechnol.* **38**, 147–150 (2020).
3. Blei, D. M., Kucukelbir, A. & McAuliffe, J. D. Variational Inference: A Review for Statisticians. *J. Am. Stat. Assoc.* **112**, 859–877 (2017).
4. Tipping, M. E. & Bishop, C. M. Probabilistic principal component analysis. *J. R. Stat. Soc. Series B Stat. Methodol.* **61**, 611–622 (1999).
5. Bishop, C. Bayesian pca. *Adv. Neural Inf. Process. Syst.* **11**, (1998).
6. Tipping, M. E. Probabilistic visualisation of high-dimensional binary data. *Adv. Neural Inf. Process. Syst.* 592–598 (1998).
7. Khan, M. E., Marlin, B. M., Bouchard, G. & Murphy, K. P. Variational bounds for mixed-data factor analysis. *Adv. Neural Inf. Process. Syst.* 1108–1116 (2010).
8. Abid, A., Zhang, M. J., Bagaria, V. K. & Zou, J. Contrastive Principal Component Analysis. *arXiv [stat.ML]* (2017).
9. Li, D., Jones, A. & Engelhardt, B. Probabilistic Contrastive Principal Component Analysis. *arXiv [stat.ME]* (2020).
10. Abdi, H. Partial Least Squares (PLS) Regression. (2003).

11. Jolliffe, I. T. A Note on the Use of Principal Components in Regression. *J. R. Stat. Soc. Ser. C Appl. Stat.* **31**, 300–303 (1982).
12. Abdi, H. Partial least squares regression and projection on latent structure regression (PLS Regression). *Wiley Interdiscip. Rev. Comput. Stat.* **2**, 97–106 (2010).
13. Lindgren, F. & Rännar, S. Alternative Partial Least-Squares (PLS) Algorithms. in *3D QSAR in Drug Design: Recent Advances* (eds. Kubinyi, H., Folkers, G. & Martin, Y. C.) 105–113 (Springer Netherlands, Dordrecht, 1998).
14. Westland, J. C. Partial Least Squares Path Analysis. in *Structural Equation Models: From Paths to Networks* (ed. Westland, J. C.) 17–38 (Springer International Publishing, Cham, 2019).
15. Sæbø, S., Almøy, T., Flatberg, A., Aastveit, A. H. & Martens, H. LPLS-regression: a method for prediction and classification under the influence of background information on predictor variables. *Chemometrics Intellig. Lab. Syst.* **91**, 121–132 (2008).
16. Hardoon, D. R., Szedmak, S. & Shawe-Taylor, J. Canonical correlation analysis: an overview with application to learning methods. *Neural Comput.* **16**, 2639–2664 (2004).
17. Hotelling, H. RELATIONS BETWEEN TWO SETS OF VARIATES. *Biometrika* **28**, 321–377 (1936).
18. Abdi, H., Guillemot, V., Eslami, A. & Beaton, D. Canonical Correlation Analysis. in *Encyclopedia of Social Network Analysis and Mining* (eds. Alhajj, R. & Rokne, J.) 1–16 (Springer New York, New York, NY, 2017).
19. Klami, A., Virtanen, S. & Kaski, S. Bayesian Canonical correlation analysis. *J. Mach. Learn. Res.* **14**, 965–1003 (2013).
20. Argelaguet, R. *et al.* Multi‐Omics Factor Analysis—a framework for unsupervised integration of multi‐omics data sets. *Mol. Syst. Biol.* **14**, e8124 (2018).
21. Argelaguet, R. *et al.* MOFA+: a statistical framework for comprehensive integration of multi-modal single-cell data. *Genome Biol.* **21**, 111 (2020).
22. Boileau, P., Hejazi, N. S. & Dudoit, S. Exploring high-dimensional biological data with sparse contrastive principal component analysis. *Bioinformatics* **36**, 3422–3430 (2020).

23. Zhang, Z. *et al.* Recovery of biological signals lost in single-cell batch integration with CellANOVA. *Nat. Biotechnol.* 1–17 (2024).
24. Korsunsky, I. *et al.* Fast, sensitive and accurate integration of single-cell data with Harmony. *Nat. Methods* **16**, 1289–1296 (2019).
25. Butler, A., Hoffman, P., Smibert, P., Papalexi, E. & Satija, R. Integrating single-cell transcriptomic data across different conditions, technologies, and species. *Nat. Biotechnol.* **36**, 411–420 (2018).
26. Leek, J. T., Johnson, W. E., Parker, H. S., Jaffe, A. E. & Storey, J. D. The sva package for removing batch effects and other unwanted variation in high-throughput experiments. *Bioinformatics* **28**, 882–883 (2012).
27. Leek, J. T. & Storey, J. D. Capturing heterogeneity in gene expression studies by surrogate variable analysis. *PLoS Genet.* **3**, 1724–1735 (2007).
28. Gagnon-Bartsch, J. A. & Speed, T. P. Using control genes to correct for unwanted variation in microarray data. *Biostatistics* **13**, 539–552 (2012).
29. Risso, D., Ngai, J., Speed, T. P. & Dudoit, S. Normalization of RNA-seq data using factor analysis of control genes or samples. *Nat. Biotechnol.* **32**, 896–902 (2014).
30. Molania, R. *et al.* Removing unwanted variation from large-scale RNA sequencing data with PRPS. *Nat. Biotechnol.* **41**, 82–95 (2023).
31. Madrigal, A., Lu, T., Soto, L. M. & Najafabadi, H. S. A unified model for interpretable latent embedding of multi-sample, multi-condition single-cell data. *bioRxiv* 2023.08.15.553327 (2023) doi:10.1101/2023.08.15.553327.
