# Supplementary material for: Joint modeling of cellular heterogeneity and condition effects with scPCA in single-cell RNA-seq
Source: Commun Biol. 2026 Feb 4;9:492. doi: 10.1038/s42003-026-09651-6 (PMC13057030; doi:10.1038/s42003-026-09651-6)
Supplement: Supplementary file 3 — Description of Additional Supplementary Files [file 42003_2026_9651_MOESM3_ESM.pdf]

Description of Additional Supplementary File

File name: Supplementary Data 1
Description: Source data for Main Figures.

File name: Supplementary Data 2
Description: Source data for Supplementary Figures.
